# Discovering golden ratio in the world’s first five-agent network in ancient China

**DOI:** 10.1038/s41598-023-46071-6

**Published:** 2023-10-30

**Authors:** Ciann-Dong Yang

**Affiliations:** Department of Aeronautics and Astronautics, National Cheng Kung University, Tainan, Taiwan

**Keywords:** Engineering, Mathematics and computing

## Abstract

The world’s first five-agent network, also called Wuxing network in ancient China, had been fully established in the second century BC. Surprisingly, the key to cracking the operation of Wuxing network is the golden ratio, the world’s most astonishing number originating from ancient Greece. Wuxing network is composed of five agents located on the vertices of a pentagon such that adjacent agents cooperate with each other, while spaced-apart agents oppose each other. Although it was proposed more than 2000 years ago, it is still an unparalleled network operation protocol. This article reveals the role of the golden ratio in the balance and stability of Wuxing network, and demonstrates how to detect the golden ratio experimentally in Wuxing electronic circuits and in Wuxing formation flight of drones.

## Introduction

Wuxing Network^1–3^ and the golden ratio^4,5^ each originated independently from the early days of Chinese and Greek civilizations. So far they have diverse applications in their respective fields, but the relationship between them is unknown to the world. The main reason is that Wuxing network has always lacked a rigorous mathematical model to describe its operation, so that its relationship with the golden ratio has never been discovered. In this article, we use the multi-agent network theory to establish a mathematical model describing the operation of Wuxing network. From this model, we find that since the emergence of Wuxing network, the golden ratio has been hidden in it, controlling its balance and stability. Practical methods to detect the golden ratio in Wuxing network are proposed in terms of electronic circuits and formation flight of drones. More significantly, through the geometric meaning of the golden ratio, we can extend the ancient five-agent Wuxing network to obtain a general N-agent network and the accompanying general golden ratio.

**Figure 1:**
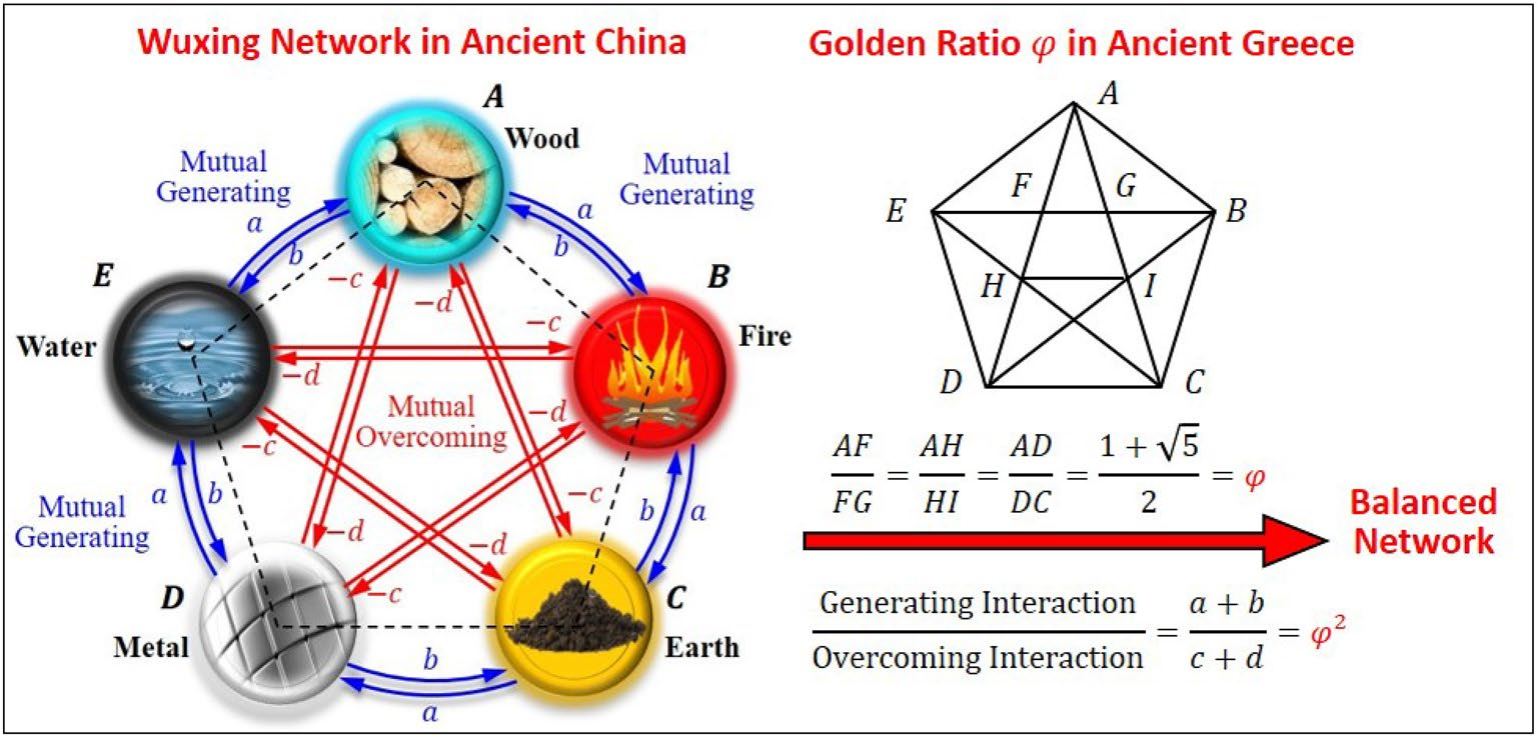
Both Wuxing network and the golden ratio are related to the regular pentagon. The golden ratio $\varphi$ refers to the ratio of the diagonal length to the side length in a regular pentagon. In Wuxing network, agents connected by the sides of the pentagon cooperate with each other (mutual generating with weights $a$ and $b$), while agents connected diagonally oppose each other (mutual overcoming with weights $-c$ and $-d$). It is discovered that the generating and overcoming interactions are balanced if the ratio $(a+b)/(c+d)$ is equal to the squared golden ratio ${\varphi }^{2}$.

Wuxing network is a natural philosophical thought in ancient China^1–3,6^. It matured in the second century BC to unify the laws of nature and human body with worldwide applications to seemingly disparate fields such as dynastic transitions^7^, geomancy^8^, astrology^9^, traditional Chinese medicine^10^, painting^11^, music^12^, military strategy^13^, and martial arts^14^. The system described by Wuxing network contains five elements, which are represented pictographically by five natural substances: wood, fire, earth, metal, and water, located on the vertices of a pentagon as shown in Fig. 1. Due to this pictographic correspondence, Wuxing network is often misunderstood as a classification of natural matter, similar to the Four Elements doctrine of early Greek philosophy^15^. In fact, the original concept of Wuxing philosophy is to express the inter-conversion between five components or units in a system through the analogy with the five natural substances. In other words, Wuxing network is concerned with the growth and decline between the five components, rather than the physical entities represented by the five components.

Wuxing network has long been widely used in different systems, whose size and attributes vary widely. In these systems, the five elements in Wuxing pictograph no longer refer to five specific substances, but are abstracted into five characteristics, five phases, or five trends of the system. These different connotations of Wuxing philosophy have evolved into a variety of different agents representing the five elements. Wuxing network has the most typical agents in history in three broad categories. The agents in the sky are the five planets of Jupiter, Mars, Saturn, Venus, and Mercury; the agents on the ground are the five seasons of spring, summer, long summer, autumn, and winter; the agents in the human body are the five organs of liver, heart, spleen, lung and kidney. It is based on the interactions among these three categories of agents of Wuxing network that traditional Chinese medicine explains the causes of human diseases and proposes the methods of medical treatment^10^.

The operation of Wuxing pictograph depends on the mutual interaction between the agents, regardless of the physical entities represented by the agents. From the classification of modern science, Wuxing pictograph actually represents a multi-agent network^16,17^, in which the number of agents is five. The agent nature of Wuxing network allows it to transcend the limitations of the physical systems and to apply to completely unrelated fields. Wuxing network proposed the world's first network operation protocol that adjacent agents cooperate with each other (mutual generating), while spaced-apart agents compete against each other (mutual overcoming), as shown in Fig. 1. Wuxing operation protocol is not created by one person at one time and one place. Its creation is the evolution result of a long history that began in the Western Zhou Dynasty (1046–771 BC). A complete description of Wuxing operation protocol appeared for the first time in the book: *Luxuriant Dew of the Spring and Autumn Annals*, edited by Dong Zhongshu (192–104 BC, a Han dynasty Chinese philosopher, politician, and writer).

The Wuxing philosophy advocates that Wuxing network with mixed cooperative and antagonistic interactions can reach the state of harmony, in which two opposing interactions are balanced. This subject is particularly important in the current situation of limited global resources. The operation of Wuxing network shows us how to achieve a harmonic relationship between resource cooperation and competition. In the past two thousand years, many Chinese philosophers employed Wuxing network consisting of agents with different nature to demonstrate the spirit of harmony. However, due to the lack of a mathematical model that can correctly describe the operation of Wuxing network, the balance and harmony of Wuxing network remains only a philosophical point of view. In view of the scientific demand for traditional Chinese medicine, whose essential logic is based on Wuxing philosophy, a few Chinese literatures^18–20^ have established preliminary mathematical models for Wuxing network. Without the consideration of modern multi-agent network theory and graph theory^6^, the mathematical models established so far are unable to manifest the main features of Wuxing Network.

## Results

### The first encounter with the golden ratio in Wuxing network

What makes us curious is why Wuxing network and the golden ratio derived from different Eastern and Western cultural thoughts are related? The first reason that comes to mind is, perhaps because they are all related to regular pentagons. As shown in Fig. 1, the golden ratio refers to the ratio of the diagonal length to the side length of a regular pentagon. On the other hand, the positions of the five agents of Wuxing network can also be connected to form a regular pentagon. However, this apparent reason did not fully respond to our curiosity, because what Wuxing network describes is not a static geometric pattern, but a dynamic process. We must further explore the possible role of the golden ratio from the dynamic behavior of Wuxing network.

The operation of Wuxing network obeys the rule that the neighboring agents cooperate with each other (mutual generating), while the spaced-apart agents compete with each other (mutual overcoming). Cooperative interaction reduces the difference between agents, while antagonistic interaction expands the difference between agents. So when the interactions of cooperation and antagonism occur at the same time, is the difference between the five agents smaller or larger? It is in the process of analyzing this problem that we first discovered the vague shadow of the golden ratio in Wuxing network. In order to quantify the interaction, we let $a>0$ and $b>0$ be the cooperative weights, and their sum $a+b$ represents the intensity of the cooperative interaction, as shown in Fig. 1. Similarly, we let $-c<0$ and $-d<0$ be the antagonistic weights, and their magnitude sum $c+d$ represents the intensity of the antagonistic interaction.

A very intuitive inference is that when the cooperative intensity $a+b$ is equal to the antagonistic intensity $c+d$, Wuxing network should be in a balanced state, that is, the difference between the five agents is neither increased nor decreased. However, the computation results show that this intuitive inference is wrong. The actual situation is that when $a+b=c+d$, the difference between the five agents will become larger and larger over time. In other words, in this case, the antagonistic effect is still stronger than the cooperative effect. Obviously, the cooperative intensity $a+b$ must be greater than the antagonistic intensity $c+d$ for Wuxing network to reach a balanced state. Therefore, we gradually increase the intensity ratio $(a+b)/(c+d)$ from 1 and monitor the dynamic response of Wuxing network to search for a balanced state. Sure enough, when $(a+b)/(c+d)$ increases to a critical value approximately equal to 2.618, Wuxing network reaches the expected balanced state. If this threshold is further exceeded, Wuxing network will assume a state of consensus, that is, the difference between the five agents will decrease to zero over time. This is our first encounter with the golden ratio in the operation of Wuxing network, as described in Fig. 1. Later in the theoretical verification, we learned that this critical value of 2.618 turned out to be an approximation of the squared golden ratio ${\varphi }^{2}$.

### The theoretical evidence of the golden ratio in Wuxing network

The critical ratio $(a+b)/(c+d)=2.618$ forms the watershed of the dynamic response of Wuxing network. This particular number 2.618 arouses our interest in further investigation of Wuxing network. The agent nature of Wuxing network allows us to replace the five natural substances with five abstract agents, represented by five symbols A, B, C, D, E, as long as their interactions follow the Wuxing network’s operation protocol. Different from the existing multi-agent networks, the agents in Wuxing network cannot be divided into groups such that two agents belonging to the same group are friends with cooperative interaction and two agents belonging to different groups are enemies with antagonistic interaction. Existing discussions on multi-agent networks, such as balance, stability, consensus, and control, etc., are aimed at cooperative networks^21,22^, bipartite networks^23–26^, or multi-party networks^27^, which all cannot be applied directly to Wuxing network.

Fortunately, the special structure of Wuxing network allows us to analyze its dynamic behavior in an analytical way. Let the quantitative indices of the five agents be represented respectively by ${x}_{A}$, ${x}_{B}$,${x}_{C}$, ${x}_{D}$, and ${x}_{E}$. The interchanges between ${x}_{i}$ under the influence of the cooperative and antagonistic interactions can be described by a linear equation $\dot{X}={\mathbb{A}}_{5}X$, with $X={\left[{x}_{A}, {x}_{B}, {x}_{C}, {x}_{D}, {x}_{E}\right]}^{T}$. The system matrix ${\mathbb{A}}_{5}$, which can be expressed in terms of the cooperative weights $a$ and $b$, and the antagonistic weights $-c$ and $-d$, determines the dynamic behavior of Wuxing network. The stability of Wuxing network depends on whether the real part of the dominant eigenvalue of ${\mathbb{A}}_{5}$, $\mathrm{Re}({\uplambda }_{\mathrm{domi}}\left({\mathbb{A}}_{5}\right))$, is positive or negative. The boundary of stability is formed by the condition: $\mathrm{Re}\left({\uplambda }_{\mathrm{domi}}\left({\mathbb{A}}_{5}\right)\right)=0$, from which the constraint imposed on the intensity ratio can be found analytically as:

1 $$\frac{a+b}{c+d}={\varphi }^{2}.$$

Here we formally encounter the golden ratio $\varphi =(1+\sqrt{5})/2$ in Wuxing network. The squared golden ratio ${\varphi }^{2}=1+\varphi \approx 2.618$ is just the critical value we found previously in the numerical analysis of Wuxing network.

**Figure 2:**
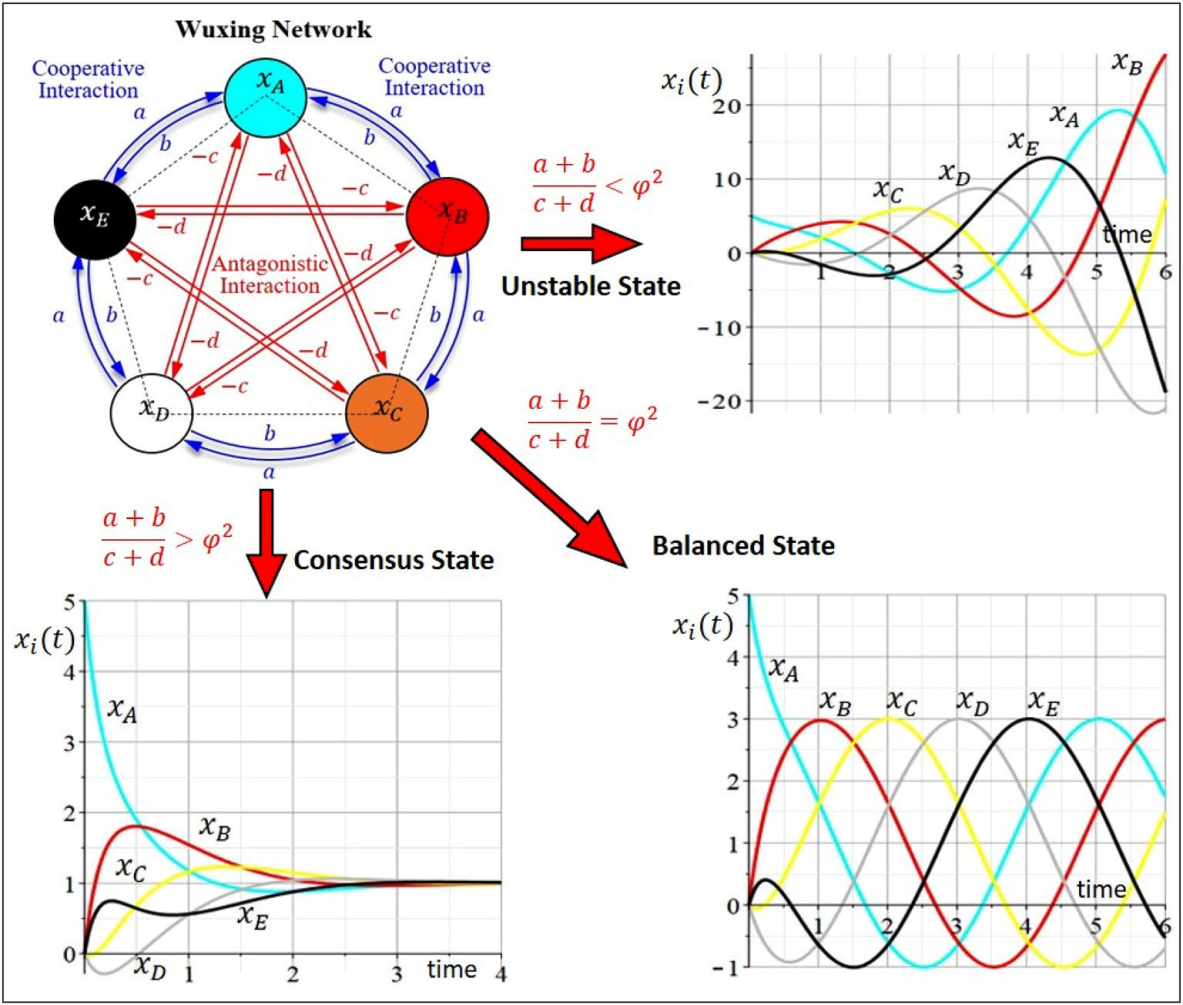
Three dynamic states of Wuxing network corresponding to three regions of the intensity ratio. When the intensity ratio $(a+b)/(c+d)$ is less than ${\varphi }^{2}$, Wuxing network exhibits unstable dynamic behavior. When the intensity ratio is equal to ${\varphi }^{2}$, Wuxing network reaches a balanced state, where ${x}_{i}(t)$ presents a harmonic oscillation. When the intensity ratio is greater than ${\varphi }^{2}$, Wuxing network enters a consensus state, where ${x}_{i}(t)$ converges to the same value.

Depending on the intensity ratio $(a+b)/(c+d)$, Wuxing network presents three different dynamic behaviors, as shown in Fig. 2. When the intensity ratio is lower than ${\varphi }^{2}$, Wuxing network is unstable with divergent ${x}_{i}(t)$. When the intensity ratio is equal to ${\varphi }^{2}$, Wuxing network is balanced such that ${x}_{i}(t)$ neither diverges nor converges, but presents harmonic oscillation. When the intensity ratio is greater than ${\varphi }^{2}$, Wuxing network enters the state of consensus, where ${x}_{i}(t)$ converges to the same value. Equation (1) provides the theoretical evidence that the golden ratio is an inseparable part of Wuxing network. However, this evidence has never been discovered during the historic development of Wuxing network.

### Detecting golden ratio in Wuxing electronic circuit

**Figure 3:**
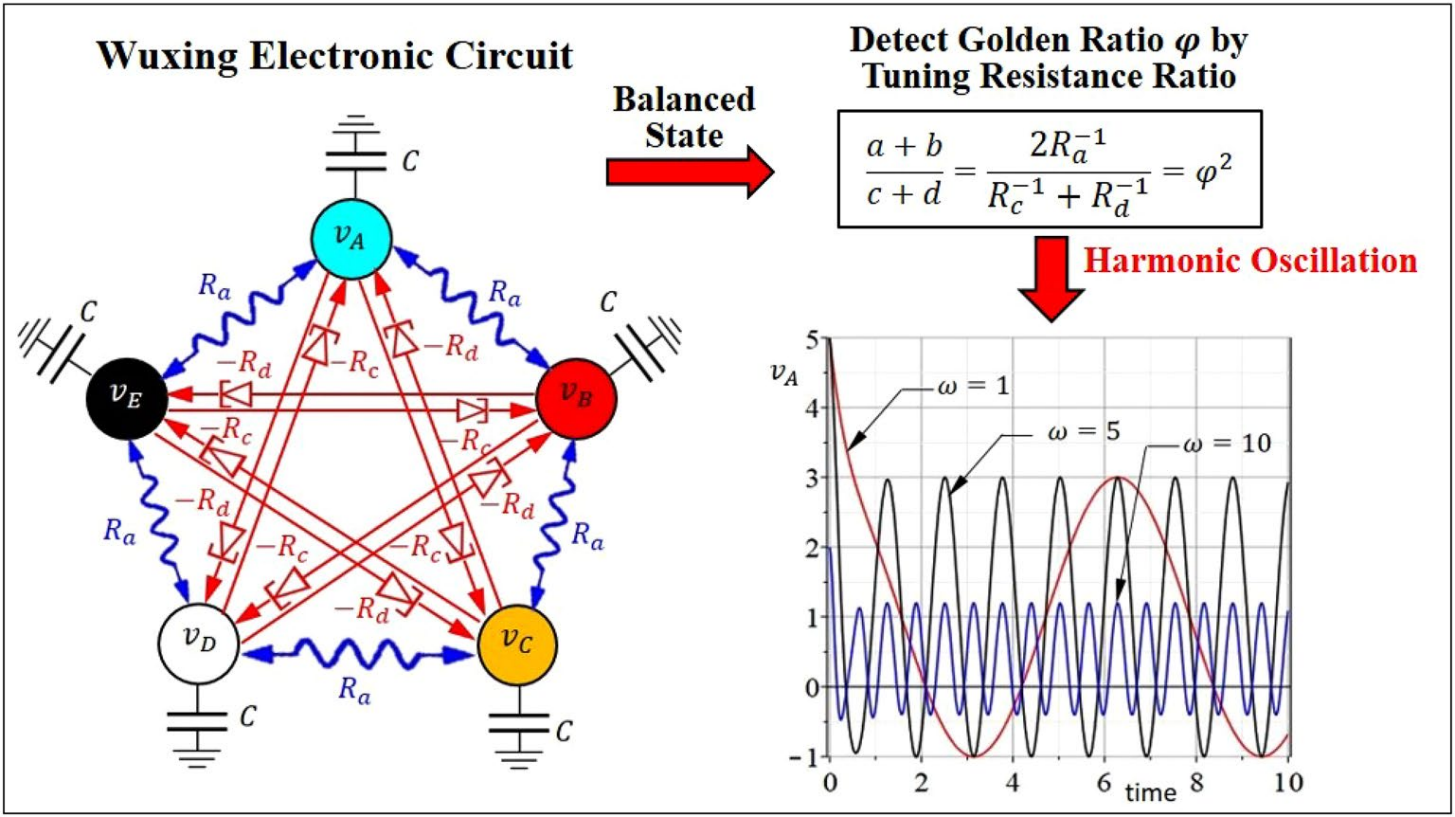
Detecting golden ratio in Wuxing electronic circuit. The negative resistances $-{R}_{c}<0$ and $-{R}_{d}<0$ of two resonant tunneling diodes are used to implement the antagonistic interaction in Wuxing network, while the normal resistor ${R}_{a}>0$ implements the cooperative interaction. The golden ratio can be detected experimentally by tuning the value of $(a+b)/(c+d)=2{R}_{a}^{-1}/({R}_{c}^{-1}+{R}_{d}^{-1})$ until the node’s voltage exhibits harmonic oscillation. The tuned point of $2{R}_{a}^{-1}/({R}_{c}^{-1}+{R}_{d}^{-1})$ then gives the squared golden ratio ${\varphi }^{2}$.

The existence of the golden ratio in Wuxing network can be detected experimentally by realizing Wuxing network in terms of electronic circuits. The resulting Wuxing circuit has five nodes arranged in such a manner that the adjacent nodes are connected by positive resistance ${R}_{a}$ to exhibit cooperative interaction with weights $a=b={R}_{a}^{-1}>0$, while the spaced-apart nodes are connected by negative resistances $-{R}_{c}$ and $-{R}_{d}$ to exhibit antagonistic interaction with weights $-c=-{R}_{c}^{-1}<0$ and $-d=-{R}_{d}^{-1}<0$, as shown in Fig. 3.

Negative resistance is also called negative differential resistance^28^, which refers to the characteristic that the voltage of some circuits or electronic components decreases when the current in a certain range increases. In general, when the current increases, the voltage also increases, but the negative resistance exhibits the opposite of the normal resistance. At present, there is no single electronic component that can exhibit negative resistance in all working ranges. However, some diodes, such as resonant tunneling diode^29^, have negative resistance in a specific working range. The red component symbol in Fig. 3 represents the resonant tunneling diode operating in the region with negative resistance.

Wuxing circuit shown in Fig. 3 is a modern realization of Wuxing network in Fig. 2, and the dynamic behaviors of the letter can be synthesized practically by the electronic signals of the former. The analogy between Figs. 2 and 3 provides us an experimental method to detect the golden ratio by tuning the resistance ratio $(a+b)/(c+d)=2{R}_{a}^{-1}/({R}_{c}^{-1}+{R}_{d}^{-1})$ until the node’s voltage exhibits harmonic oscillation. The tuned point of $2{R}_{a}^{-1}/({R}_{c}^{-1}+{R}_{d}^{-1})$ then exactly gives the squared golden ratio ${\varphi }^{2}$. When operating at the tuned point, Wuxing electronic circuit behaves like a harmonic oscillator, whose oscillation frequency $\omega$ can be freely specified by adjusting the relative magnitude of ${R}_{c}$ to ${R}_{d}$.

### Detecting golden ratio in Wuxing-formation flight

**Figure 4:**
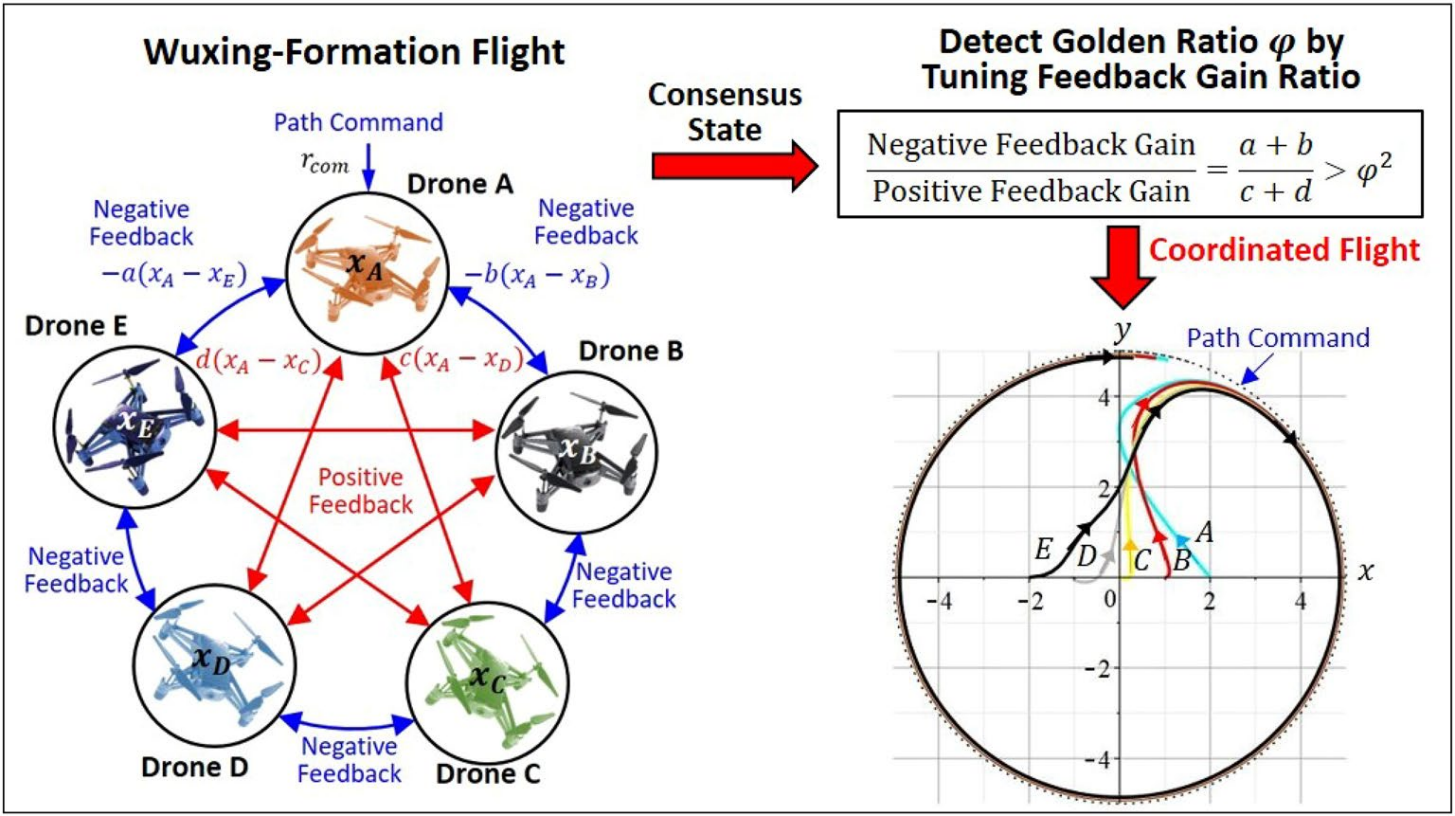
Detecting golden ratio in Wuxing-formation flight. The five drones play the role of autonomous agents in Wuxing network in such a way that adjacent drones form a negative feedback loop (blue lines), while space-apart drones form a positive feedback loop (red lines). The coordinated flight of the five drones requires that the ratio of the negative feedback gain to the positive feedback gain must be greater than the squared golden ratio.

The autonomous agents of Wuxing network could be five drones, five self-driving cars or five intelligent robots, as long as the agent’s behavior can comply with the operation protocol of Wuxing network. Figure 4 realizes the operation of Wuxing network by the formation flight of five drones. The flight network consisting five drones implements the hybrid interactions of Wuxing network through five automatic control units with sensing, computing and driving capabilities.

Different from the current formation flight for which all the drones are friends^30,31^, Wuxing formation flight has an extraordinary property that the adjacent drones with cooperative interaction are friends and they tend to fly together, while the spaced-apart drones with antagonistic interaction are enemies and they tend to fly apart. From the viewpoint of feedback control theory^32^, the cooperative interaction in Wuxing network is equivalent to negative feedback control with gains $a$ and $b$ (blue loops in Fig. 4), and the antagonistic interaction is equivalent to positive feedback control with gains $c$ and $d$ (red loops in Fig. 4). Positive feedback increases the coordination error between the drones, while negative feedback reduces the coordination error. Therefore, the ancient Wuxing network raises a challenge to the modern control theory about how to ensure the system’s stability if negative feedback and positive feedback coexist within the system. However, Wuxing network had already solved this control problem by itself with the help of its intrinsic partner: the golden ratio.

In order to achieve the formation flight, the five drones have to reach the state of consensus, where the coordination error between them converges to zero. The analogy between Figs. 2 and 4 indicates that the state of consensus is attained by requiring that the ratio of the negative feedback gain $a+b$ to the positive feedback gain $c+d$ must be greater than the squared golden ratio ${\varphi }^{2}$. The right part of Fig. 4 shows that drone A receives a circular path command ${r}_{com}$ and leads the other drones to flight coordinately along the path command, once the condition of consensus $(a+b)/(c+d)>{\varphi }^{2}$ is satisfied. Hence, ${\varphi }^{2}$ is the critical value of the feedback-gain ratio $(a+b)/(c+d)$, below which the five drones fly apart and above which the five drones fly coordinately.

### General Wuxing network and general golden ratio

**Figure 5:**
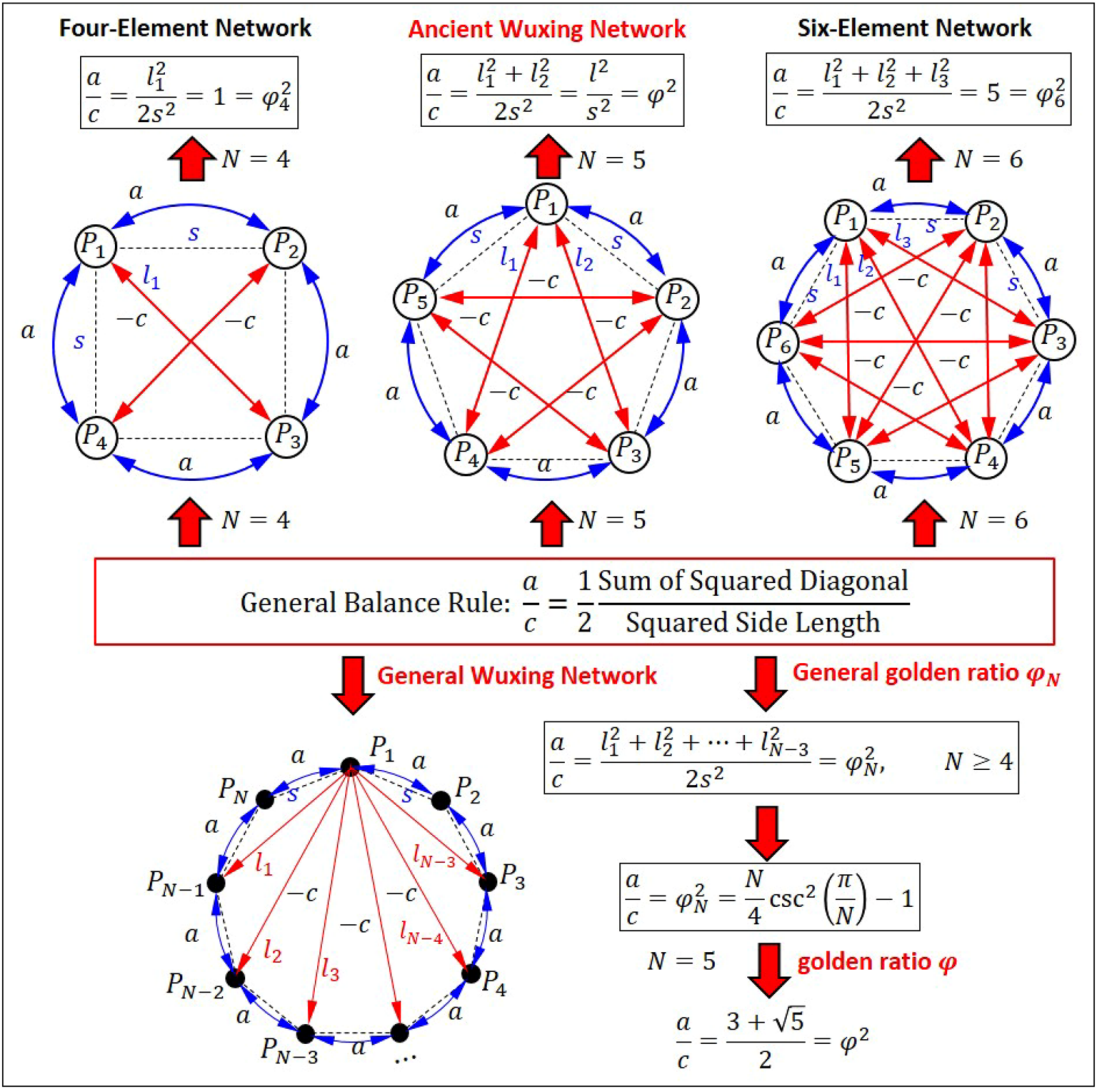
General Wuxing network and general golden ratio. The agents of the N-element Wuxing network locate on the vertices of a regular N-sided polygon. The cooperative agents are connected by the sides of the polygon with weight $a$, while the antagonistic agents are connected by the diagonals of the polygon with weight $-c$. The general balance rule is given by $a/c={\varphi }_{N}^{2}$, where ${\varphi }_{N}$ is the general golden ratio defined for the N-element Wuxing network. When $N=5$, the general rule reduces to $a/c={\varphi }^{2}$, as already shown in Fig. 2.

The ancient Wuxing network only had five agents. This limitation made it difficult to be applied to modern networks. With the clue given by the golden ratio, we can extend Wuxing network to a network with N elements (N agents) without changing its operation protocol. As shown in Fig. 5, the N agents to be considered are arranged on a circle at equal intervals to form a regular N-sided polygon. The relationship between two agents is defined to be adjacent, if their connection line is a side of the polygon, and is defined to be spaced apart, if their connection line is a diagonal of the polygon. In the network with $\mathrm{N}=3$, the three agents are all adjacent to each other, and there is no antagonistic relationship. Therefore, for the N-element network to have both cooperative and antagonistic relationships, the minimum value of N is four, as shown in Fig. 5.

In the N-element Wuxing network, the only agents adjacent to a certain agent ${P}_{1}$ are its left and right agents, which have cooperative interactions with agent ${P}_{1}$. The remaining $\mathrm{N}-3$ agents are all separated from agent ${P}_{1}$, and have antagonistic interactions with agent ${P}_{1}$. When N increases, the number of cooperative interactions with agent ${P}_{1}$ remains unchanged at 2, but the number of antagonistic interactions is getting larger. Therefore, in order to maintain the balance of the network, the cooperative intensity $a+b$ must be more and more greater than the antagonistic intensity $c+d$, as the number of agent increases. The special structure of Wuxing network with $\mathrm{N}=5$ gives us a clue to determine the balance condition of the N-element network by using a simple geometric rule. To simplify the analysis, we assume that the action and reaction between two agents are equal in magnitude, i.e., $a=b$ and $c=d$. This simplification does not lose its generality, because the balance of Wuxing network depends on the values of $a+b$ and $c+d$, rather than their individual values.

The clue comes from the geometric meaning of the golden ratio $\varphi =l/s$, where $l$ is the diagonal length and $s$ is the side length of a regular pentagon. The balance condition of the five-element Wuxing network, $(a+b)/(c+d)={\varphi }^{2}={l}^{2}/{s}^{2}$, now can be rewritten as $2a{s}^{2}=2c{l}^{2}$. We recall that in the five-element Wuxing network, the diagonal $l$ is weighted by $c$ to denote the antagonistic intensity and the side length $s$ is weighted by $a$ to denote the cooperative intensity. Therefore, the condition $2a{s}^{2}=2c{l}^{2}$ can be interpreted as the balance between the total cooperative intensity and the total antagonistic intensity. For the N-element Wuxing network, there are still two sides with length $s$ connecting to a certain element ${P}_{1}$, but there are $N-3$ diagonals, with length ${l}_{1}$, ${l}_{2}$, $\cdots$, ${l}_{N-3}$, connecting to ${P}_{1}$, as shown in Fig. 5. Therefore, the extension of the balance condition $2a{s}^{2}=2c{l}^{2}$ to the N-element Wuxing network becomes $2a{s}^{2}=c({l}_{1}^{2}+{l}_{2}^{2}+\cdots +{l}_{N-3}^{2})$, which can be restated as the following general rule:

2 $$\frac{a}{c}=\frac{{\overline{{P }_{3}{P}_{1}}}^{2}+{\overline{{P }_{4}{P}_{1}}}^{2}+\cdots +{\overline{{P }_{N-1}{P}_{1}}}^{2}}{{\overline{{P }_{2}{P}_{1}}}^{2}+{\overline{{P }_{N}{P}_{1}}}^{2}}=\frac{{l}_{N-3}^{2}+{l}_{N-4}^{2}+\cdots +{l}_{1}^{2}}{2{s}^{2}}={\varphi }_{N}^{2}.$$

This equality can be shown to be equivalent to the stability condition: $\mathrm{Re}\left({\uplambda }_{\mathrm{domi}}\left({\mathbb{A}}_{N}\right)\right)=0$, where ${\mathbb{A}}_{N}$ is the system matrix of the N-element Wuxing network. The number ${\varphi }_{N}$ in Eq. (2) is the general golden ratio defined for the N-element Wuxing network, which has an analytical expression ${\varphi }_{N}^{2}=(N/4){\text{csc}}^{2}(\pi /N)-1$. When $N=5$, ${\varphi }_{N}$ becomes the golden ration $\varphi$ and Eq. (2) reduces to Eq. (1). If $\varphi$ is replaced by ${\varphi }_{N}$, the results in Figs. 2 to 4 can all be applied to the N-element Wuxing network.

## Discussion

Wuxing network embodies the golden mean of Chinese culture through the balance between cooperative and competitive interactions. The golden mean, which appeared both in the Confucian philosophy and Aristotelian philosophy, is the desirable middle between two extremes, one of excess and the other of deficiency. When the antagonistic interaction is greater than the cooperative interaction, Wuxing network diverges into disorder. Conversely, when the cooperative interaction is greater than the antagonistic interaction, Wuxing network converges into consensus. The golden mean of Wuxing network is to maintain the balance of the two interactions, so that the network achieves a balanced state that is neither divergent nor convergent. In this article, we used the language of multi-agent network theory to express how Wuxing network presents the golden mean between the two extremes. We found that the condition required by Wuxing network to achieve the philosophical golden mean is given by the mathematical golden mean, i.e. the golden ratio $\varphi =(1+\sqrt{5})/2$, which is recognized as the most astonishing number in the world^4^. The time when the golden ratio appeared in ancient Greek is roughly the time when Wuxing network appeared in ancient Chinese. Both have been widely used in many different fields for more than two thousand years. With the bridge established in this paper, the irrelevant developments of the golden ratio and Wuxing network in different fields will hopefully be integrated.

Since Wuxing philosophy is the foundation of traditional Chinese medicine^33^, the main application of the mathematical model established in this article for the Wuxing network is in traditional Chinese medicine to make it expressible in the language of modern science. As for the engineering field, Wuxing network is helpful for analyzing the balance and stability of the engineering networks when there are both cooperative and antagonistic relationships in the network structure.

From the perspective of modern multi-agent network systems, the ancient Wuxing network naturally has many limitations. This is because the time and space background when it was proposed is completely different from now. Three potential limitations within the Wuxing network are as follows:

- The special network structure of Wuxing network: Wuxing network requires that adjacent agents must have a cooperative relationship, while spaced-apart agents must have an antagonistic relationship. This special structure of Wuxing network is derived from the unique traditional Chinese culture, and its characteristics are not shared by ordinary networks.
- Wuxing network is a simplified form of five-agent network: A general five-agent network has twenty weights, but Wuxing network has only four weights $a$, $b$, $c$,$d$, corresponding to generating action, generating reaction, overcoming action, and overcoming reaction, respectively. Only in this simplified form can we see the relationship between the golden ratio and the Wuxing network. However, this relationship does not exist in the general five-agent network.
- Wuxing network only allows five agents: The number of agents in Wuxing network is five, which is a counting unit originating from ancient China. This unique number of agents now becomes a limitation on the application of Wuxing network. The final part of this article has extended the five-agent Wuxing network to the N-agent Wuxing network; however, the latter is still not a general N-agent network, because it possesses the inherent property of Wuxing network that adjacent agents cooperate mutually, while spaced-apart agents compete mutually.

In the possible follow-up research on Wuxing network, how to maintain its unique cultural connotation while taking into account its application in modern science has become the first topic we must face.

## Methods

### Modeling Wuxing network as a weighted graph

**Figure 6:**
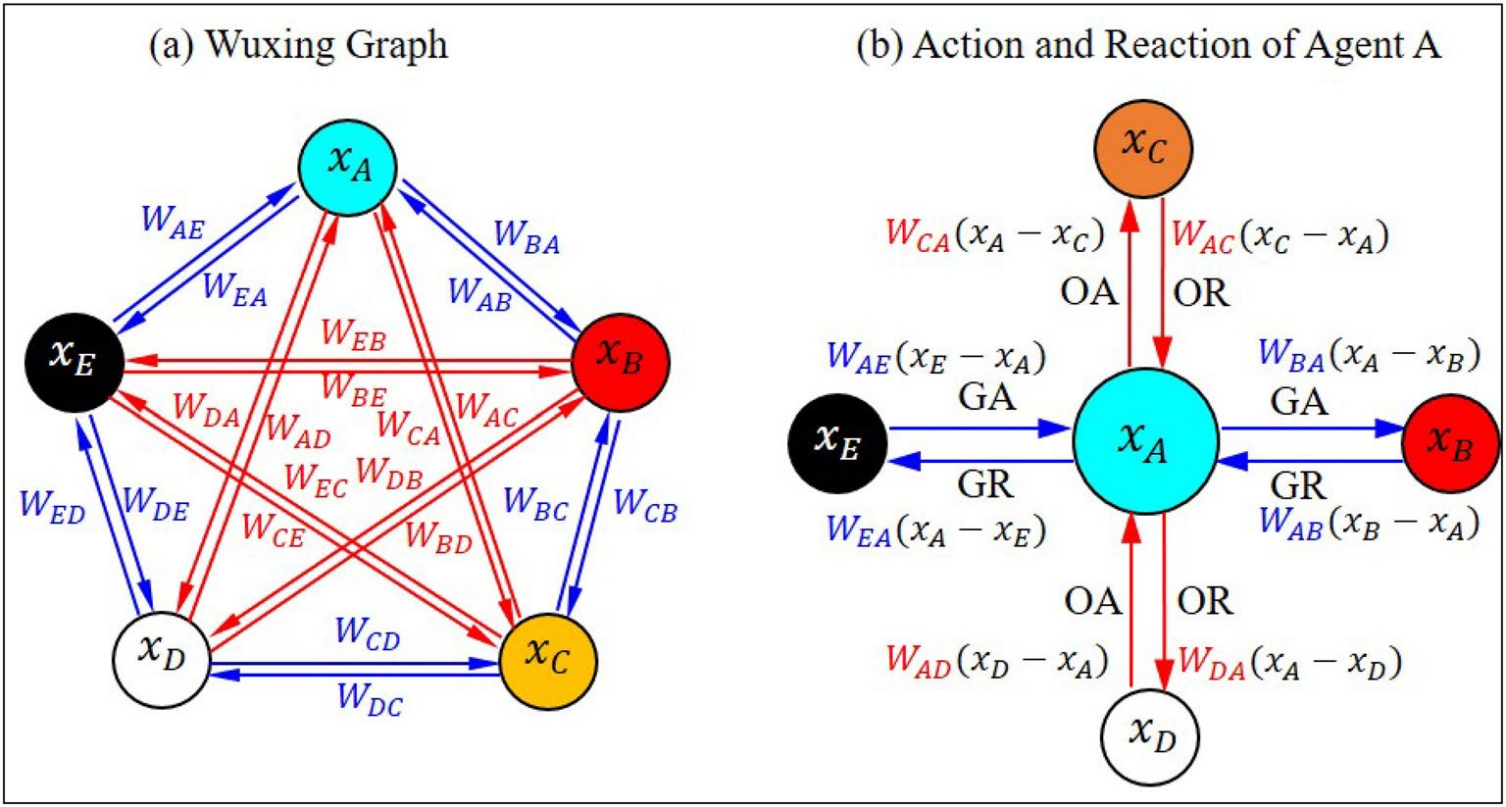
The Wuxing graph and the interactions between agent A and the other four agents. (**a**) Wuxing network can be represented as a weighted graph with 5 vertices and 20 edges, where the edge pointing from agent $j$ to agent $i$ is weighted by ${W}_{ij}$. The blue lines denote the generating interaction between adjacent agents with weight ${W}_{ij}>0$, while the red lines denote the overcoming interaction between spaced-apart agents with weight ${W}_{ij}<0$. (**b**) The four edges connected inward to agent A represent, respectively, generating action (GA), generating reaction (GR), overcoming action (OA), and overcoming reaction (OR), which are applied to agent A by the other four agents. The four edges connected outward from agent A represent the four effects imposed by Agent A on the other four agents.

The operation of Wuxing pictograph can be summarized into the following three principles:

- Two Agents’ relative position determines the attribute of their relationship. Whether the relationship is cooperative or antagonistic is not determined by the physical property of the agents, but by their relative positions in the network, as regulated by Wuxing operation protocol: adjacent agents generate each other, while spaced-apart agents overcome each other.
- Action is always accompanied by reaction. Generating action and overcoming action cannot be carried out freely, but must pay the price, which is their accompanying reactions. Generating action and its accompanying reaction constitute the cooperative interaction, while overcoming action and its reaction constitute the antagonistic interaction, as shown in Fig. 6b.
- Two agents’ relative magnitude determines the direction and intensity of their interactions. The directions of the generating action and the overcoming action cannot be prescribed in advance, but are spontaneously determined by the relative magnitude of the agents.

The first operation principle allows us to replace the five natural substances with five abstract agents, represented by five symbols A, B, C, D, E, as long as their interactions follow the Wuxing operation protocol. The second operation principle implies that the interactions between the agents are two-way effects, containing the forward actions and the accompanying reactions. The third operation principle tells us how to determine the direction and intensity of the interaction in an algebraic way. Assuming that the quantitative indices of five abstract agents are represented, respectively, by ${x}_{A}$, ${x}_{B}$,${x}_{C}$, ${x}_{D}$, and ${x}_{E}$, then the sign of ${x}_{i}-{x}_{j}$ automatically determines whether the direction of the action is $i\to j$ or $j\to i$. Meanwhile, the magnitude of ${x}_{j}-{x}_{i}$ weighted by ${W}_{ij}$ determines the intensity of the interaction, where ${W}_{ij}>0$, if the interaction is cooperative, and ${W}_{ij}<0$, if the interaction is antagonistic.

**Figure 7:**
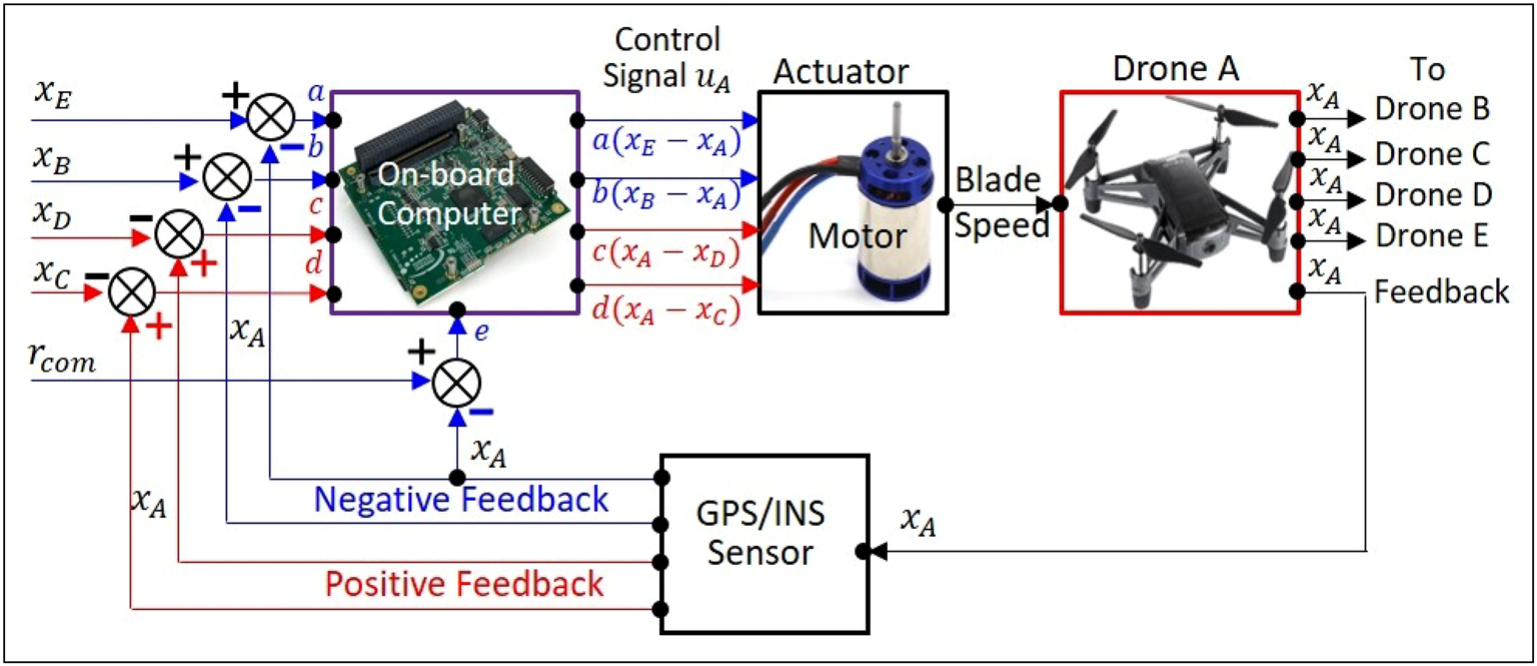
The control block diagram of the formation flight based on Wuxing network. To implement the operation protocol of Wuxing network, each drone is equipped with a sensor, an on-board computer, and an actuator. The blue lines represent the negative feedback loop produced by the cooperative interaction with feedback gain $a$ and $b$, and the red lines represent the positive feedback loop produced by the antagonistic interaction with feedback gain $c$ and $d$. Negative feedback makes the five drones fly in formation, while positive feedback tends to disintegrate the formation flight. Whether the drones fly coordinately or fly apart depends on the relative magnitude of $(a+b)/(c+d)$ to the squared golden ratio ${\varphi }^{2}$.

According to the above three operation principles of Wuxing network, we can define Wuxing graph as following. Wuxing graph is a weighted graph $\mathcal{G}=(V,E)$ having five vertices $V=\left\{{x}_{A},{x}_{B},{x}_{C},{x}_{D},{x}_{E}\right\}$ arranged in a pentagon with 20 edges, i.e., $\left|E\right|=20$. The edge ${E}_{j\to i}$ is weighted by ${W}_{ij}$, which is positive if ${E}_{j\to i}$ is a side of the pentagon, and is negative if ${E}_{j\to i}$ is a diagonal of the pentagon, as shown in Fig. 7a.

For each vertex of Wuxing graph, there are 8 edges connected to it, of which four edges are connected outward and four edges are connected inward. In other words, the degree of each vertex of Wuxing graph is 8, with in-degree and out-degree both equal to 4. At each moment, the quantitative index ${x}_{i}$ for each agent is simultaneously influenced by the indices of the other four agents, so the indices of the five agents actually change from time to time, i.e. ${x}_{i}$ is a function of time, denoted by ${x}_{i}(t)$.

Like agent A in Fig. 6b, each agent of Wuxing network is subject to four effects: generating action (GA), generating reaction (GR), overcoming action (OA), and overcoming reaction (OR), coming from the other four agents, respectively; meanwhile, each agent in turn applies actions or reactions to the other four agents, as summarized in Supplementary Table S1. Adding the four effects in each row of the table, we obtain the time change rate of each agent as follows:

3 $${\dot{x}}_{i}=\sum_{j\ne i}{W}_{ij}\left({x}_{j}\left(t\right)-{x}_{i}(t)\right), \, i=A,B, C, D, E.$$

Since the type of interactions in Wuxing network depends on the relative positions of the agents, it has nothing to do with their absolute positions, and no agent is in a particularly dominant position. Wuxing graph shown in Fig. 6a reveals the fact that rotating the graph clockwise or counterclockwise does not change the operation of Wuxing network. In other words, the interaction of Wuxing graph is of rotation invariance. With this kind of rotation invariance, there are only four differences between the 20 weights in Eq. (3):

- GA weights: $${W}_{AE}={W}_{BA}={W}_{CB}={W}_{DC}={W}_{ED}=a>0$$.
- GR weights: $${W}_{EA}={W}_{AB}={W}_{BC}={W}_{CD}={W}_{DE}=b>0$$.
- OA Weights: $${W}_{CA}={W}_{EC}={W}_{BE}={W}_{DB}={W}_{AD}=-c<0$$.
- OR Weighs: $${W}_{AC}={W}_{CE}={W}_{EB}={W}_{BD}={W}_{DA}=-d<0$$.

In Wuxing network, all the GA weights are equal to $a$ and all the GR weights are all equal to $b$, and they are collectively called cooperative weights, as shown by the blue lines in Fig. 6a. On the other hand, all the OA weights are equal to $-c$ and all the OR weights are all equal to $-d$, and they are collectively called antagonistic weights, as shown by the red lines in Fig. 6a. In this way the impact of the 20 weights in Eq. (3) can be simplified into an analysis of the four representative weights, $a$, $b$, $c$, and $d$, denoting the intensities of GA, GR, OA, and OR, respectively. With the four representative weights, Eq. (3) is reduced to the following form

4a $${\dot{x}}_{A}=a\left({x}_{E}-{x}_{A}\right)+b\left({x}_{B}-{x}_{A}\right)-c\left({x}_{D}-{x}_{A}\right)-d\left({x}_{C}-{x}_{A}\right),$$

4b $${\dot{x}}_{B}=a\left({x}_{A}-{x}_{B}\right)+b\left({x}_{C}-{x}_{B}\right)-c\left({x}_{E}-{x}_{B}\right)-d\left({x}_{D}-{x}_{B}\right),$$

4c $${\dot{x}}_{C}=a\left({x}_{B}-{x}_{C}\right)+b\left({x}_{D}-{x}_{C}\right)-c\left({x}_{A}-{x}_{C}\right)-d\left({x}_{E}-{x}_{C}\right),$$

4d $${\dot{x}}_{D}=a\left({x}_{C}-{x}_{D}\right)+b\left({x}_{E}-{x}_{D}\right)-c\left({x}_{B}-{x}_{D}\right)-d\left({x}_{A}-{x}_{D}\right),$$

4e $${\dot{x}}_{E}=a\left({x}_{D}-{x}_{E}\right)+b\left({x}_{A}-{x}_{E}\right)-c\left({x}_{C}-{x}_{E}\right)-d\left({x}_{B}-{x}_{E}\right).$$

which can be recast into a matrix form as

5 $$\left[ {\begin{array}{*{20}c} {\dot{x}_{A} } \\ {\dot{x}_{B} } \\ {\dot{x}_{C} } \\ {\dot{x}_{D} } \\ {\dot{x}_{E} } \\ \end{array} } \right] = \left[ {\begin{array}{*{20}c} \sigma & b & { - d} & { - c} & a \\ a & \sigma & b & { - d} & { - c} \\ { - c} & a & \sigma & b & { - d} \\ { - d} & { - c} & a & \sigma & b \\ b & { - d} & { - c} & a & \sigma \\ \end{array} } \right]\left[ {\begin{array}{*{20}c} {x_{A} } \\ {x_{B} } \\ {x_{C} } \\ {x_{D} } \\ {x_{E} } \\ \end{array} } \right] \Rightarrow \dot{X} = {\mathbb{A}}_{5} X = - L\left( {\mathcal{G}} \right)X,$$

where $\sigma =-(a+b-c-d)$ is the negative sum of the four weights. It can be seen that the system matrix ${\mathbb{A}}_{5}$ is just the negative Laplacian matrix $L(\mathcal{G})$ of Wuxing graph. Solving the five simultaneous differential equations in Eq. (5) provides us with the time evolution of the quantitative index ${x}_{i}(t)$ of the five agents.

### The role of golden ratio in Wuxing network

There are three states existing in Wuxing network, i.e. the state of consensus, the state of balance, and the state of instability. Of significance is that which of the states will happen depends on the golden ratio $\varphi =(1+\sqrt{5})/2$. Different weights in Eqs. (4a–4e) lead to different system matrices ${\mathbb{A}}_{5}$ in Eq. (5), and result in different network’s dynamic behaviors. In order to know the dynamic behaviors of the weighted Wuxing network under different settings of weights, eigenvalues of the system matrix ${\mathbb{A}}_{5}$ must be evaluated first. The eigenvalues of ${\mathbb{A}}_{5}$ are defined as the roots of the characteristic polynomial of ${\mathbb{A}}_{5}$:

6 $$\mathrm{det}\left(\mathrm{\lambda I}-{\mathbb{A}}_{5}\right)=\uplambda \left(\uplambda -{\uplambda }_{2}\right)\left(\uplambda -{\uplambda }_{3}\right)\left(\uplambda -{\uplambda }_{4}\right)\left(\uplambda -{\uplambda }_{5}\right)=0.$$

The special structure of the matrix ${\mathbb{A}}_{5}$ manifests that it has a zero eigenvalue ${\uplambda }_{1}=0$ and two pairs of complex conjugate eigenvalues, which can be expressed as explicit functions of the four weights $a$, $b$, $c$, and $d$ as

7a $${\lambda }_{2,3}=-\frac{1}{2}\left[\alpha \left(a+b\right)-\beta \left(c+d\right)\right]\pm \frac{1}{2}\left[\sqrt{\beta }\left(a-b\right)-\sqrt{\alpha }\left(c-d\right)\right]i,$$

7b $${\lambda }_{\mathrm{4,5}}=-\frac{1}{2}\left[\beta \left(a+b\right)-\alpha \left(c+d\right)\right]\pm \frac{1}{2}\left[\sqrt{\alpha }\left(a-b\right)+\sqrt{\beta }\left(c-d\right)\right]i,$$

where $i=\sqrt{-1}$ is the imaginary number, and the two constants $\alpha$ and $\beta$ are defined as

8 $$\alpha =\frac{5-\sqrt{5}}{2}, \beta =\frac{5+\sqrt{5}}{2}.$$

Equations (7a) and (7b) gives a one-to-one relationship between the four non-zero eigenvalues and the four weights. It can be seen that the four weights determine the eigenvalues of the system matrix ${\mathbb{A}}_{5}$ in a group of $a$ and $b$ and a group of $c$ and $d$. More precisely, ${a}_{+}=a+b$ and ${c}_{+}=c+d$ constitute the real parts of the eigenvalues and determine the converging speed of the network, while ${a}_{-}=a-b$ and ${c}_{-}=c-d$ constitute the imaginary parts of the eigenvalues and determine the oscillation frequency of the network. We recall that the weights $a$ and $b$ correspond to the cooperative interaction between adjacent agents, while $c$ and $d$ correspond to the antagonistic interaction between spaced-apart agents. Therefore, the eigenvalues of the system matrix ${\mathbb{A}}_{5}$ is determined by two types of relationships: the relationship between adjacent agents and the relationship between spaced-apart agents. Thus the discovered eigen-structure of the system matrix ${\mathbb{A}}_{5}$ is consistent with the ancient Wuxing philosophy, which divides the relationship between the five elements into adjacent and spaced-apart dichotomy.

From the linear system theory, the stability of the weighted Wuxing network requires that the real part of the non-zero eigenvalues of ${\mathbb{A}}_{5}$ must be negative. This requirement gives a lower bound on the weight ratio $(a+b)/(c+d)$ in terms of the golden ratio. From Eqs. (7a) and (7b), we find that the real part of ${\lambda }_{\mathrm{2,3}}$ is greater than the real part of ${\lambda }_{\mathrm{4,5}}$ by noting

9 $$\mathrm{Re}\left({\lambda }_{\mathrm{2,3}}\right)-\mathrm{Re}\left({\lambda }_{\mathrm{4,5}}\right)=\frac{1}{2}\left(\beta -\alpha \right)\left(a+b+c+d\right)>0$$

where $\beta -\alpha =\sqrt{5}$ and the four parameters $a$, $b$, $c$, and $d$ are all positive. Therefore, the requirement that the real parts of the non-zero eigenvalues must be negative is equivalent to $\mathrm{Re}\left({\lambda }_{\mathrm{2,3}}\right)<0$, i.e.

10 $$-\frac{1}{2}\left[\alpha \left(a+b\right)-\beta \left(c+d\right)\right]<0 \Rightarrow \frac{{a}_{+}}{{c}_{+}}=\frac{a+b}{c+d}>\frac{\beta }{\alpha }={\varphi }^{2}.$$

where $\beta /\alpha =(3+\sqrt{5})/2$ is just equal to the squared golden ratio ${\varphi }^{2}$, which gives the lower bound on the ration ${a}_{+}/{c}_{+}$ to guarantee the stability of the weighted Wuxing network.

It is worth noting that the golden ratio is exactly the ratio between the diagonal length and the side length of a regular pentagon. This relationship provides a valuable clue that allows us to extend the five-element Wuxing network to a generalized Wuxing network with N-element.

The solutions ${x}_{i}(t)$, $i=A, B, \cdots , E$, to Eq. (5) can be expressed in terms of ${\lambda }_{\mathrm{2,3}}={\sigma }_{\mathrm{2,3}}\pm {\omega }_{\mathrm{2,3}}i$ and ${\lambda }_{\mathrm{4,5}}={\sigma }_{\mathrm{4,5}}\pm {\omega }_{\mathrm{4,5}}i$ as

11 $${x}_{i}\left(t\right)={C}_{i}+{A}_{i}{e}^{{\sigma }_{\mathrm{2,3}}t}\mathrm{sin}({\omega }_{\mathrm{2,3}}t+{\theta }_{i})+{B}_{i}{e}^{{\sigma }_{\mathrm{4,5}}t}\mathrm{sin}\left({\omega }_{\mathrm{4,5}}t+{\phi }_{i}\right),$$

where the constant ${C}_{i}$ originates from the stationary mode ${e}^{{\lambda }_{1}t}$ with ${\lambda }_{1}=0$, and ${A}_{i}$, ${B}_{i}$, ${\theta }_{i}$, ${\phi }_{i}$ are constants determined from the initial conditions of ${x}_{i}\left(0\right)$. As $t\to \infty$, the agent’s quantitative index ${x}_{i}\left(t\right)$ in Eq. (11) approaches three different states, depending on the relative magnitude of ${a}_{+}/{c}_{+}$ to ${\varphi }^{2}$.

(a) The unstable state: ${a}_{+}/{c}_{+}<{\varphi }^{2}$

In case of ${a}_{+}/{c}_{+}<{\varphi }^{2}$, we have ${\sigma }_{\mathrm{2,3}}>0$, which implies ${e}^{{\sigma }_{\mathrm{2,3}}t}\to \infty$, as $t\to \infty$. Therefore, Eq. (11) shows that all ${x}_{i}\left(t\right)$ diverge to infinity, i.e. ${x}_{i}\left(t\right)\to \infty$, as $t\to \infty$.

(b) The balanced state: ${a}_{+}/{c}_{+}={\varphi }^{2}$

The critical condition ${a}_{+}/{c}_{+}={\varphi }^{2}$ is the boundary between the state of consensus and the state of instability. We note that ${a}_{+}/{c}_{+}=(a+b)/(c+d)$ represents the ratio between the sum of cooperative weights and the sum of antagonistic weights. In case of ${a}_{+}/{c}_{+}>{\varphi }^{2}$, the cooperative interaction in Wuxing network is larger than the antagonistic interaction, causing the network to converge to the state of consensus. In case of ${a}_{+}/{c}_{+}<{\varphi }^{2}$, the antagonistic interaction is larger and causes the network to diverge. When the equality ${a}_{+}/{c}_{+}={\varphi }^{2}$ is established, the cooperative interaction and the antagonistic interaction are balanced. Under this condition, Wuxing network neither converges nor diverges, but exhibits a behavior with harmonic oscillation. Substituting ${a}_{+}/{c}_{+}={\varphi }^{2}$ into Eqs. (7a) and (7b), we obtain ${\sigma }_{\mathrm{2,3}}=0$ and ${\sigma }_{\mathrm{4,5}}<0$, which is then used in Eq. (11) to yield

12 $$\underset{t\to \infty }{\mathrm{lim}}{x}_{i}\left(t\right)={C}_{i}+{A}_{i}\mathrm{sin}({\omega }_{\mathrm{2,3}}t+{\theta }_{i}), \, i=A, B, \cdots , E.$$

This is just a harmonic wave with mean ${C}_{i}$, amplitude ${A}_{i}$, frequency ${\omega }_{\mathrm{2,3}}$, and phase ${\theta }_{i}$.

(c) The consensus state: ${a}_{+}/{c}_{+}>{\varphi }^{2}$

In case of ${a}_{+}/{c}_{+}>{\varphi }^{2}$, we have ${\sigma }_{\mathrm{4,5}}<{\sigma }_{\mathrm{2,3}}<0$, which implies ${e}^{{\sigma }_{\mathrm{2,3}}t}\to 0$ and ${e}^{{\sigma }_{\mathrm{4,5}}t}\to 0$, as $t\to \infty$. Hence, from Eq. (11) we have

13 $$\underset{t\to \infty }{\mathrm{lim}}{x}_{i}\left(t\right)={C}_{i}, \, i=A, B, \cdots , E,$$

where ${C}_{i}$ is the steady-state value of ${x}_{i}\left(t\right)$.

From the above analysis, we find that the balance of the two opposite interactions in Wuxing network does not occur at ${a}_{+}/{c}_{+}=1$, but at ${a}_{+}/{c}_{+}={\varphi }^{2}$. Why must the ratio of ${a}_{+}$ to ${c}_{+}$ be exactly equal to ${\varphi }^{2}$ in order to maintain the balance of Wuxing network? The answer is that the direction of the cooperative interaction weighted by ${a}_{+}$ is along the side of a regular pentagon, while the direction of the antagonistic interaction weighted by ${c}_{+}$ is along its diagonal. The reason for ${a}_{+}/{c}_{+}={\varphi }^{2}$ becomes obvious, if we notice that the golden ratio $\varphi$ is just the ratio of the diagonal to the side of a regular pentagon.

### Detecting golden ratio in Wuxing electronic circuits

A straightforward realization of Wuxing network is given by electronic circuits, where the quantitative indices ${x}_{i}(t)$ correspond to the voltages of five nodes, the cooperative weights $a$ and $b$ between adjacent agents corresponds to the positive resistances ${R}_{a}$ and ${R}_{b}$, and the antagonistic weights $-c$ and $-d$ between spaced-apart agents corresponds to the negative resistances ${-R}_{c}$ and $-{R}_{d}$, as shown in Fig. 3. The voltages of the five nodes are represented, respectively, by ${v}_{A}$, ${v}_{B}$, ${v}_{C}$, ${v}_{D}$, and ${v}_{E}$. The capacitances at the five nodes are all set to $C$, and the charges stored in the capacitors are denoted by ${Q}_{A}$, ${Q}_{B}$, ${Q}_{C}$, ${Q}_{D}$, and ${Q}_{E}$. According to the law of conservation of charge, the change rate of the charge for each node can be expressed as follows:

14a $${\dot{Q}}_{A}=C{\dot{v}}_{A}=\frac{{v}_{E}-{v}_{A}}{{R}_{a}}+\frac{{v}_{B}-{v}_{A}}{{R}_{a}}+\frac{{v}_{D}-{v}_{A}}{-{R}_{c}}+\frac{{v}_{C}-{v}_{A}}{-{R}_{d}},$$

14b $${\dot{Q}}_{B}=C{\dot{v}}_{B}=\frac{{v}_{A}-{v}_{B}}{{R}_{a}}+\frac{{v}_{C}-{v}_{B}}{{R}_{a}}+\frac{{v}_{E}-{v}_{B}}{-{R}_{c}}+\frac{{v}_{D}-{v}_{B}}{-{R}_{d}},$$

14c $${\dot{Q}}_{C}=C{\dot{v}}_{C}=\frac{{v}_{B}-{v}_{C}}{{R}_{a}}+\frac{{v}_{D}-{v}_{C}}{{R}_{a}}+\frac{{v}_{A}-{v}_{C}}{-{R}_{c}}+\frac{{v}_{E}-{v}_{C}}{-{R}_{d}},$$

14d $${\dot{Q}}_{D}=C{\dot{v}}_{D}=\frac{{v}_{C}-{v}_{D}}{{R}_{a}}+\frac{{v}_{E}-{v}_{D}}{{R}_{a}}+\frac{{v}_{B}-{v}_{D}}{-{R}_{c}}+\frac{{v}_{A}-{v}_{D}}{-{R}_{d}},$$

14e $${\dot{Q}}_{E}=C{\dot{v}}_{E}=\frac{{v}_{D}-{v}_{E}}{{R}_{a}}+\frac{{v}_{A}-{v}_{E}}{{R}_{a}}+\frac{{v}_{C}-{v}_{E}}{-{R}_{c}}+\frac{{v}_{B}-{v}_{E}}{-{R}_{d}},$$

where ${R}_{a}>0$ is the resistance of a normal resistor and $-{R}_{c}<0$ and $-{R}_{d}<0$ are the resistances of two resonant tunneling diodes operating in the range of negative resistance. By redefining the above parameters as

15 $${(C{R}_{a})}^{-1}=a>0, \, -{\left(C{R}_{c}\right)}^{-1}=-c<0, \, -{\left(C{R}_{d}\right)}^{-1}=-d<0.$$

Equations (14a–14e) can be recast into a familiar form:

16a $${\dot{v}}_{A}=a\left({v}_{E}-{v}_{A}\right)+a\left({v}_{B}-{v}_{A}\right)-c\left({v}_{D}-{v}_{A}\right)-d\left({v}_{C}-{v}_{A}\right),$$

16b $${\dot{v}}_{B}=a\left({v}_{A}-{v}_{B}\right)+a\left({v}_{C}-{v}_{B}\right)-c\left({v}_{E}-{v}_{B}\right)-d\left({v}_{D}-{v}_{B}\right),$$

16c $${\dot{v}}_{C}=a\left({v}_{B}-{v}_{C}\right)+a\left({v}_{D}-{v}_{C}\right)-c\left({v}_{A}-{v}_{C}\right)-d\left({v}_{E}-{v}_{C}\right),$$

16d $${\dot{v}}_{D}=a\left({v}_{C}-{v}_{D}\right)+a\left({v}_{E}-{v}_{D}\right)-c\left({v}_{B}-{v}_{D}\right)-d\left({v}_{A}-{v}_{D}\right),$$

16e $${\dot{v}}_{E}=a\left({v}_{D}-{v}_{E}\right)+a\left({v}_{A}-{v}_{E}\right)-c\left({v}_{C}-{v}_{E}\right)-d\left({v}_{B}-{v}_{E}\right).$$

Comparing Eqs. (16a–16e) with Eqs. (4a–4e), we can see that the circuit Eqs. (16a–16e) is just the mathematical model for Wuxing network with $a=b$. The five eigenvalues of the system matrix associated with Eqs. (16a–16e) can be found from Eqs. (7a) and (7b) with $a=b$ as ${\lambda }_{1}=0$ and

17a $${\lambda }_{\mathrm{2,3}}={\sigma }_{\mathrm{2,3}}\pm {\omega }_{\mathrm{2,3}}i=-\frac{1}{2}\left[2\alpha a-\beta \left(c+d\right)\right]\pm \frac{\sqrt{\alpha }}{2}\left(c-d\right)i,$$

17b $${\lambda }_{\mathrm{4,5}}={\sigma }_{\mathrm{4,5}}\pm {\omega }_{\mathrm{4,5}}i=-\frac{1}{2}\left[2\beta a-\alpha \left(c+d\right)\right]\pm \frac{\sqrt{\beta }}{2}\left(c-d\right)i.$$

The main application of Wuxing circuit is to work as an oscillating circuit to generate harmonic waves of various frequencies. The condition for the harmonic oscillation of Wuxing circuit is ${\sigma }_{\mathrm{2,3}}=0$, that is

18 $$\frac{2a}{c+d}=\frac{2{R}_{a}^{-1}}{{R}_{c}^{-1}+{R}_{d}^{-1}}=\frac{\beta }{\alpha }={\varphi }^{2}.$$

Under this condition, we have ${\sigma }_{\mathrm{2,3}}=0$ and ${\sigma }_{\mathrm{4,5}}<0$, and Wuxing network enters the balanced state as described by Eq. (12) with the oscillation frequency given by

19 $${\omega }_{\mathrm{2,3}}=\frac{\sqrt{\alpha }}{2}\left(c-d\right).$$

Therefore, the squared golden ratio ${\varphi }^{2}$ can be detected experimentally by tuning the resistance ratio $2a/(c+d)=2{R}_{a}^{-1}/({R}_{c}^{-1}+{R}_{d}^{-1})$ until Wuxing circuit exhibits a harmonic oscillation, and the tuned point is ${\varphi }^{2}$. If a harmonic wave of frequency ${\omega }_{\mathrm{2,3}}^{*}$ is to be generated, we only need to substitute ${\omega }_{\mathrm{2,3}}^{*}$ into Eq. (19) and solve together with the harmonic condition (18) to get the required constants $c$ and $d$, i.e. the required negative resistances of two resonant tunneling diodes. Then the resulting Wuxing circuit oscillates with the prescribed frequency ${\omega }_{\mathrm{2,3}}^{*}$. Figure 3 shows that Wuxing circuit generates harmonic waves with assigned frequencies $\omega =1$, $5$ and $10$, by choosing the values of $c$ and $d$ through Eqs. (18) and (19) with $a=1$.

### Detecting golden ratio in Wuxing formation flight

In order to produce the hybrid interaction required by Wuxing network, each drone in Fig. 4 is equipped with a sensor, an on-board computer, and an actuator. Referring to the control block diagram of drone A in Fig. 7, the sensor, which is an inertial navigation system integrated with a GPS receiver, is used to measure the current position of the drone A; the onboard computer calculates the control signal $u$ based on the position errors between drone A and the other four drones; the actuator (motor) receives the control signal $u$ to change the rotating speed of the blades to adjust the attitude and flight speed of the drone in order to achieve the coordinated flight with other drones. The agent played by drone A with sensing, computing and driving functions is an automatic control unit. The flight network composed of five drones implements the hybrid interactions of Wuxing network through five automatic control units.

From the point of view of feedback control theory, the cooperative interaction in Wuxing network is equivalent to negative feedback (blue loops in Fig. 7), and the antagonistic interaction is equivalent to positive feedback (red loops). Referring to Fig. 7, after the position ${x}_{A}$ of drone A is measured by the sensing element, it is compared with ${x}_{E}$ and ${x}_{B}$ in a negative feedback manner (i.e. with $-{x}_{A}$) to generate the control signals $a({x}_{E}-{x}_{A})$ and $b({x}_{B}-{x}_{A})$. At the same time, the position ${x}_{A}$ of drone A is also compared with ${x}_{D}$ and ${x}_{C}$ in a positive feedback manner (i.e. with $+{x}_{A}$) to generate the control signals $c({x}_{A}-{x}_{D})$ and $d({x}_{A}-{x}_{C})$. In addition to the above four feedback control signals, drone A receives an input command ${r}_{com}$ from the ground command center, which is the path command it wants to track. Combining the above five items, we get the control signal ${u}_{A}$ of drone A as:

20 $${\dot{x}}_{A}={u}_{A}=a\left({x}_{E}-{x}_{A}\right)+b\left({x}_{B}-{x}_{A}\right)+c\left({x}_{A}-{x}_{D}\right)+d\left({x}_{A}-{x}_{C}\right)+w\left({r}_{com}-{x}_{A}\right).$$

The last term of ${u}_{A}$ is called the tracking error, which is the error between the position of drone A and the path command ${r}_{com}$ to be tracked. The control signals for the other drones can be derived by the same way and the combination of the five control signals gives

21 $$\left[\begin{array}{c}{\dot{x}}_{A}\\ {\dot{x}}_{B}\\ {\dot{x}}_{C}\\ {\dot{x}}_{D}\\ {\dot{x}}_{E}\end{array}\right]=\left[\begin{array}{c}{u}_{A}\\ {u}_{B}\\ {u}_{C}\\ {u}_{D}\\ {u}_{E}\end{array}\right]=-\left[\begin{array}{cc}{e}_{AE}& {e}_{AB}\\ {e}_{BA}& {e}_{BC}\\ {e}_{CB}& {e}_{CD}\\ {e}_{DC}& {e}_{DE}\\ {e}_{ED}& {e}_{EA}\end{array}\right]\left[\begin{array}{c}a\\ b\end{array}\right]+\left[\begin{array}{cc}{e}_{AE}& {e}_{AE}\\ {e}_{BA}& {e}_{AE}\\ {e}_{CB}& {e}_{AE}\\ {e}_{DC}& {e}_{AE}\\ {e}_{ED}& {e}_{AE}\end{array}\right]\left[\begin{array}{c}c\\ d\end{array}\right]+\left[\begin{array}{c}{e}_{com}\\ 0\\ 0\\ 0\\ 0\end{array}\right]={u}_{-}+{u}_{+}+{u}_{com},$$

where ${e}_{ij}={x}_{i}-{x}_{j}$ is the coordination error between drone $i$ and drone $j$, ${e}_{com}={r}_{com}-{x}_{A}$ is the command tracking error, and ${u}_{-}$ and ${u}_{+}$ correspond to negative and positive feedback signals. Except for the command signal ${u}_{com}$, Eq. (21) is identical to Eq. (4a–4e) with ${e}_{ij}={x}_{i}-{x}_{j}$. The positive feedback control ${u}_{+}$ tends to enlarge the coordination error ${e}_{ij}$ and makes the five drones depart from each other. The appearance of the positive feedback control ${u}_{+}$ indicates that Wuxing formation flight combines friendly and enemy drones, which is different from the current formation flight for which all the drones are friendly^30,31^. Therefore, formation flight based on Wuxing network is far more difficult to realize than the existing formation flight. Nevertheless, the convergence of Wuxing network under the condition $(a+b)/(c+d)>{\varphi }^{2}$ ensures that Wuxing formation flight mission can be achieved.

The analogy between Eqs. (21) and (4a–4e) indicates that the formation flight with ${e}_{ij}\to 0$, i.e., the state of consensus, is attained by requiring that the ratio of the negative feedback gain $a+b$ to the positive feedback gain $c+d$ must be greater than the squared golden ratio ${\varphi }^{2}$. Hence, the squared golden ratio ${\varphi }^{2}$ is the critical value of the feedback-gain ratio $(a+b)/(c+d)$, below which the five drones fly apart, and above which the five drones fly coordinately. By tuning the feedback gain ratio continuously and monitoring the dynamic responses of the drones, we therefore can detect ${\varphi }^{2}$ as the critical value of feedback gain ratio.

**Figure 8:**
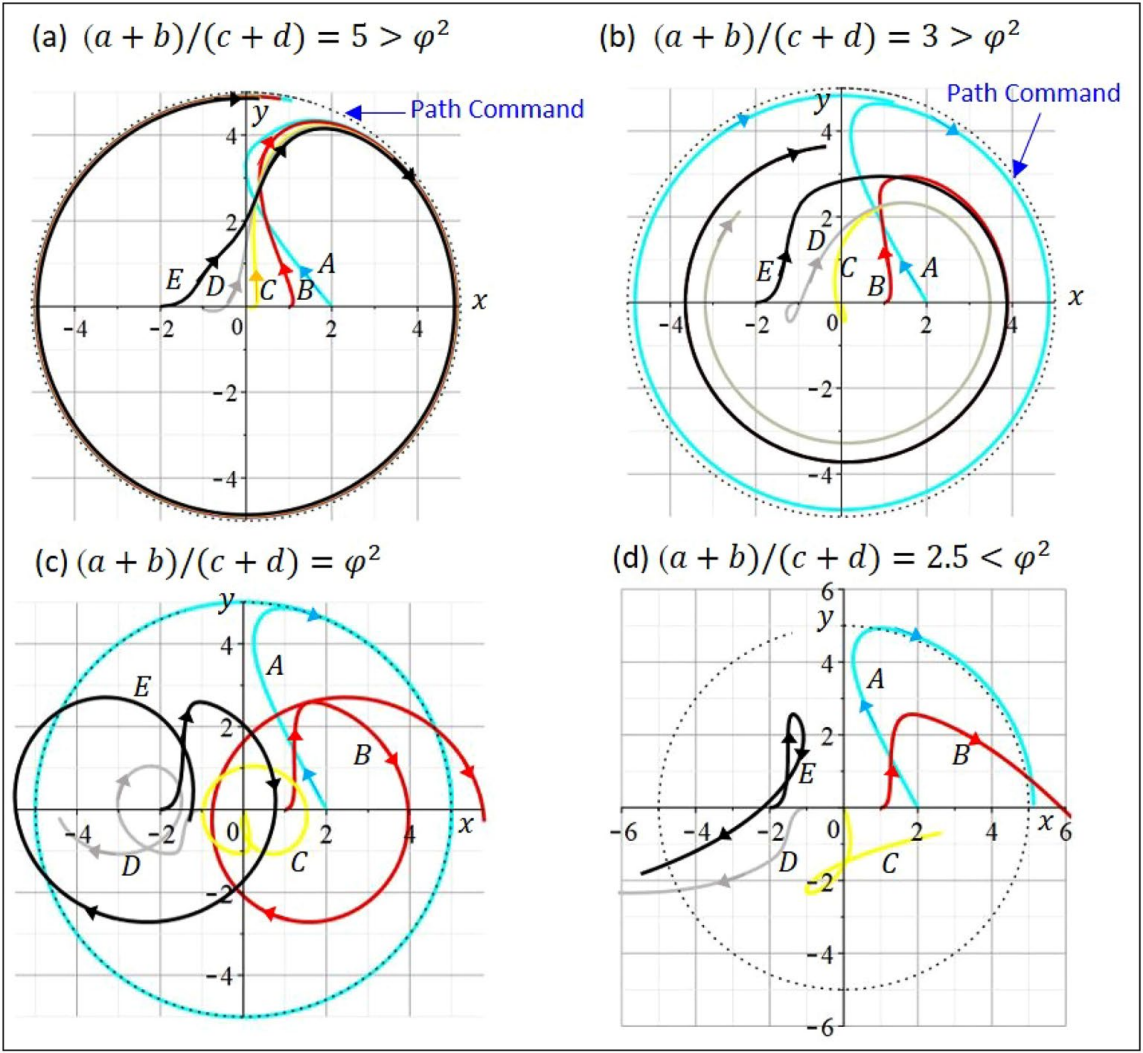
The detection of the golden ratio in Wuxing formation flight. For cases (**a**) and (**b**), the stability condition $(a+b)/(c+d)>{\varphi }^{2}$ is satisfied, and all the five drones converge to the consensus state given by the circular path command ${r}_{\mathrm{com}}=(5\mathrm{sin}\left(0.2t\right),5\mathrm{cos}\left(0.2t\right))$. Because the speed of convergence depends on the relative magnitude of $(a+b)/(c+d)$ to ${\varphi }^{2}$, the converging speed of case (**a**) to the target trajectory is faster than that of case (**b**). Case (**c**) occurs in the critical situation, in which the trajectory of the drones neither diverges nor converges to the target trajectory, but each exhibits periodic oscillations. By tuning the value of $(a+b)/(c+d)$ until the critical situation occurs, the tuned value is just the squared golden ratio ${\varphi }^{2}$. Case (**d**) corresponds to an unstable situation in which the drones fly separately, causing the formation flight to disintegrate.

Three flight patterns can be identified in Fig. 8 according to the value of $(a+b)/(c+d)$. Cases (a) and (b), which satisfy the stability condition $(a+b)/(c+d)>{\varphi }^{2}$, exhibit the formation flight that all the five drones converge to the state of consensus specified by the circular path command ${r}_{\mathrm{com}}=(5\mathrm{sin}\left(0.2t\right),5\mathrm{cos}\left(0.2t\right))$. The converging speed of case (a) is faster than that of case (b), because a larger feedback-gain ratio $(a+b)/(c+d)$ is used in the flight control for case (a). Case (c) adopts a feedback-gain ratio exactly equal to ${\varphi }^{2}$ and yields a critical flight scenario, in which the trajectory of the drones neither diverges nor converges to the target trajectory, but each exhibits periodic oscillations. Case (d) adopts a feedback-gain ratio smaller than ${\varphi }^{2}$ and produces an unstable flight pattern in which the drones fly separately to disintegrate the formation flight.

In Fig. 8, we observe that the trajectories of different drones may intersect. However, the intersection of trajectories does not mean that the drones collided together. This is because the two drones arrived at the trajectory intersection point at different times. When one drone reaches the trajectory intersection point, the other drone may not have arrived yet, or may have already left, so the two drones will not collide. The relative magnitude of the feedback-gain ratio $(a+b)/(c+d)$ to ${\varphi }^{2}$ determines which of the three flight patterns to occur. This relation allows us to determine ${\varphi }^{2}$ by tuning the feedback-gain ratio $(a+b)/(c+d)$ until the critical flight scenario appears, and then the tuned value is just the squared golden ration ${\varphi }^{2}$.

### General Wuxing network and general golden ratio

The limitation of applying Wuxing Network to modern network is that the network can only allow five agents (five elements). We can extend Wuxing network to a network with N elements without changing its operation protocol. Just like Wuxing network is accompanied by the golden ratio $\varphi$, the general N-element Wuxing network is accompanied by a general golden ratio ${\varphi }_{N}$. Referring to Fig. 5, the N agents of the general Wuxing network are arranged on a circle at equal intervals to form an N-sided regular polygon. The relationship between two agents is said to be adjacent, if their connection line is a side of the polygon, and is said to be spaced apart, if their connection line is a diagonal of the polygon.

According to the above definition of the general Wuxing network, the only agents adjacent to a certain agent ${P}_{1}$ are its left and right agents, which have cooperative interactions with agent ${P}_{1}$. The remaining $\mathrm{N}-3$ agents are all separated from agent ${P}_{1}$, which have antagonistic interactions with agent ${P}_{1}$. To simplify the stability analysis, we assume that the interactions between two agents are equal in both directions, which means that action is equal to reaction in magnitude, i.e. $a=b$ and $c=d$. This simplification does not lose its generality, because from Eq. (10), we can see that the stability of Wuxing network depends on the values of $a+b$ and $c+d$, rather than their individual values.

Among the N-element networks, Wuxing network with $\mathrm{N}=5$ is the most special, because for any agent in this network, its relationships with the other four agents happen to be two cooperative relationships and two antagonistic relationships. This seems to be the most feasible structure to achieve network balance. Except for $\mathrm{N}=5$, the number of cooperative relationships is not equal to the number of antagonistic relationships in the N-element networks. In the network with $\mathrm{N}=3$, the three agents are all adjacent to each other, and there is no antagonistic relationship. Therefore, for the N-element network to have both cooperative and antagonistic relationships, the minimum value of N is four, as shown in Fig. 5.

Similar to the derivation process of the system matrix ${\mathbb{A}}_{5}$ in Eq. (5), the system matrix corresponding to the N-element network can be expressed as follows:

22 $${\mathbb{A}}_{N}=\left[\begin{array}{ccccccccc}{\delta }_{N}& a& -c& -c& -c& \cdots & \cdots & -c& a\\ a& {\delta }_{N}& a& -c& -c& \cdots & \cdots & -c& -c\\ -c& a& {\delta }_{N}& a& -c& \cdots & \cdots & \vdots & \vdots \\ -c& -c& a& {\delta }_{N}& a& -c& \cdots & \vdots & \vdots \\ -c& -c& -c& a& {\delta }_{N}& a& -c& \vdots & \vdots \\ \vdots & \vdots & \vdots & -c& a& {\delta }_{N}& a& -c& -c\\ \vdots & \vdots & \vdots & \vdots & -c& a& {\delta }_{N}& a& -c\\ -c& -c& \cdots & \cdots & \cdots & -c& a& {\delta }_{N}& a\\ a& -c& \cdots & \cdots & \cdots & -c& -c& a& {\delta }_{N}\end{array}\right],$$

where ${\delta }_{N}=(N-3)c-2a$. When $N=5$, ${\mathbb{A}}_{N}$ reduces to the system matrix of Wuxing network given by Eq. (5) with $a=b$ and $c=d$.

The system matrix ${\mathbb{A}}_{N}$ contains $N-3$ elements of $-c$, 2 elements of $a$, and 1 element of ${\delta }_{N}$ in any vertical column and horizontal row of it. This feature ensures that ${\mathbb{A}}_{N}$ has a zero eigenvalue with the corresponding eigenvector having elements all equal to 1. Consequently, when the N-element network is stable, the quantities of its N agents, denoted by ${x}_{i}(t)$, $i=1, 2, \cdots N$, all converge to the same value, which is the state of consensus of the N-element network. However, the system matrix ${\mathbb{A}}_{N}$ given by Eq. (22) is not always stable for arbitrary $a$ and $c$. Judging the stability of the system matrix ${\mathbb{A}}_{N}$, we need to check whether all the non-zero eigenvalues of ${\mathbb{A}}_{N}$ are located in the left half plane. When N gets larger, the judgment of this condition will be more difficult. Fortunately, the special structure of Wuxing network with $\mathrm{N}=5$ gives us a clue to determine the stability of the N-element network by using a simple geometric rule.

This clue comes from the geometric meaning of the golden ratio $\varphi$. The stability condition of Wuxing network is given by $a/c>{\varphi }^{2}$, where the golden ratio $\varphi$ is just the ratio of the diagonal length $l$ to the side length $s$ of a regular pentagon, i.e., $\varphi =l/s$. Hence, the stability condition (10) of Wuxing network with $a=b$ and $c=d$ turns out to be

23 $$a{s}^{2}>c{l}^{2}.$$

We recall that in Wuxing network, the diagonal $l$ is weighted by $c$ to denote the intensity of antagonistic interaction between spaced-apart agents and the side length $s$ is weighted by $a$ to denote the intensity of cooperative interaction between adjacent agents. It is better to rewrite Eq. (23) as $2a{s}^{2}>2c{l}^{2}$ by noting that there are two diagonals and two sides connected to each vertex of the regular pentagon in Fig. 5. Therefore, the stability condition $2a{s}^{2}>2c{l}^{2}$ can be interpreted as the requirement that the total intensity of cooperative interaction must be greater than the total intensity of the antagonistic interaction. For a N-element network with $\mathrm{N}\ne 5$, we note that the number of diagonals connected to each agent is $\mathrm{N}-3$ and the number of sides connected to each agent is always two. Applying the above definition of total intensity to an N-sided polygon, we can generalize the stability condition $2a{s}^{2}>2c{l}^{2}$ to the N-element network as

24 $$2a{s}^{2}>c\sum_{k=1}^{N-3}{l}_{k}^{2} \Rightarrow \frac{a}{c}>\frac{1}{2}\sum_{k=1}^{N-3}{\left(\frac{{l}_{k}}{s}\right)}^{2}={\varphi }_{N}^{2},$$

where ${l}_{k}$, $k=1, 2, \cdots , N-3$, are the lengths of diagonals connecting to a vertex of the N-sided polygon. Letting $N=5$ in Eq. (24), and noting ${l}_{1}={l}_{2}=l$ for the pentagon in Fig. 5, we recover the stability condition $a/c>{l}^{2}/{s}^{2}={\varphi }^{2}$ for Wuxing network. The quantity ${\varphi }_{N}$ defined in Eq. (24) provides a general definition of golden ratio, which extends the geometric meaning of the golden ratio from regular pentagon to N-sided regular polygon. By expressing the diagonal length ${l}_{k}$ in terms of the side length $s$ of a N-sided regular polygon, a simple formula for the general golden ratio ${\varphi }_{N}$ can be derived as

25 $${\varphi }_{N}^{2}=\frac{N}{4}{\mathrm{csc}}^{2}\left(\frac{\pi }{N}\right)-1.$$

The verification of the stability condition (24) and the derivation of the formula (25) are given in the supplementary information. 

### Supplementary Information

Supplementary Information.

## Data Availability

All data generated or analyzed during this study are included in this published article and in the supplementary information.
