# Supplementary material for: Discovering golden ratio in the world’s first five-agent network in ancient China
Source: Sci Rep. 2023 Oct 30;13:18581. doi: 10.1038/s41598-023-46071-6 (PMC10616226; doi:10.1038/s41598-023-46071-6)
Supplement: Supplementary file 1 — Supplementary Information. [file 41598_2023_46071_MOESM1_ESM.pdf]

# Supplementary Information for

## Discovering Golden Ratio in the World's First Five-Agent Network in Ancient China

Ciann-Dong Yang

Correspondence to: cdyang@mail.ncku.edu.tw

**The historic evolution of Wuxing network.** People have long been misled by the common Wuxing pictograph, thinking that Wuxing network is composed of generating cycle and overcoming cycle with fixed sequence, i.e.,

- Generating cycle: wood → fire → earth → metal → water → wood →…
- Overcoming cycle: wood → earth → water → fire → metal → wood →…

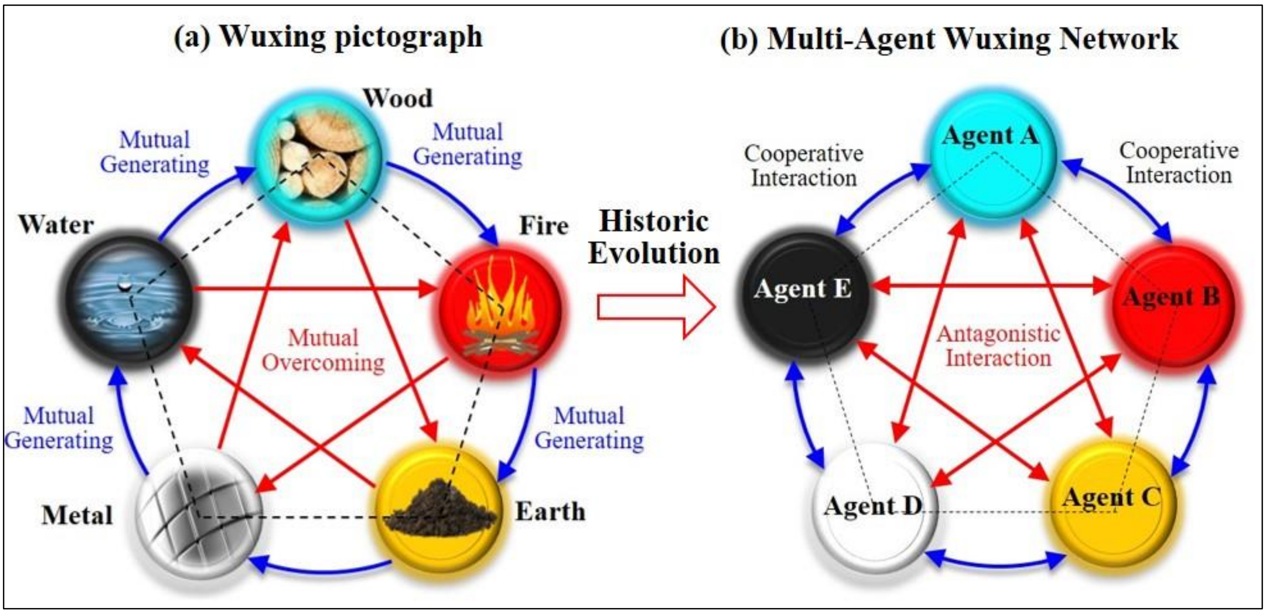


**Supplementary Fig. S1 The historic evolution from Wuxing pictograph to multi-agent Wuxing network.** (a) Wuxing pictograph in ancient China contains five natural elements, wood, fire, earth, metal, and water, located on the five vertices of a regular pentagon. The five elements in the figure are highlighted by their representative colors: cyan, red, yellow, white, and black. The inter-conversion between the elements obeys the operation principle that the adjacent elements form a generating cycle: wood → fire → earth → metal → water → wood →…, while the spaced-apart elements form an overcoming sequence: wood → earth → water → fire → metal → wood →… (b) Through the revision and improvement of many generations of ancient Chinese philosophers, Wuxing pictograph had been evolved into multi-agent Wuxing network, in which the five natural elements had been abstracted into five agents, and the unidirectional generating and overcoming cycles had been replaced by the bidirectional cooperative and antagonistic interactions between the agents.

Almost all scientific criticisms of Wuxing philosophy originate from these two fixed-direction cycles. For example, from the generating cycle: earth → metal → water →…, we get the relation that

earth generates metal. But on the other hand, from the overcoming cycle: earth → water → fire → metal → …, we get the opposite relation that earth overcomes metal. This paradoxical result stems from the failure to consider the necessary conditions for the occurrence of generating and overcoming actions. In this regard, Mozi (470-391 BC, a Chinese philosopher during the Hundred Schools of Thought period) already pointed out that the overcoming sequence in Wuxing network does not happen naturally, but must be accompanied by appropriate conditions. Mozi's statement means that in the event of agent A overcoming agent C, it only occurs if the quantity of A is greater than the quantity of C. Similar to Mozi's opinion, Sun Tzu's *Art of War*[13] mentions that which of the five elements is the object to be overcome is not permanent. Besides Mozi and Sun Tzu, many other philosophers in different dynasties, especially Chinese medicine practitioners[33], proposed amendments to the operation of Wuxing pictograph. Eventually, the Wuxing pictograph was evolved into the multi-agent Wuxing network, wherein the five natural elements were abstracted into five agents, and the unidirectional generating and overcoming cycles were replaced by the bidirectional cooperative and antagonistic interactions between the agents.

**Modeling Wuxing Network as a Weighted Graph.** We use agent A as an example to explain how the change of $x_A(t)$ is affected by the actions and reactions applied to A by the other four agents according to the three operation principles of Wuxing network.

- Agent A ↔ agent E: Agent A and agent E are adjacent (Fig. 6a) so that they generate each other according to the first operation principle. If $x_E > x_A$, agent E generates agent A and causes $x_A$ to increase. This is the generating action (GA) applied to A by E, and according to the third operation principle, the change rate of $x_A$ can be expressed as $W_{AE}(x_E - x_A) > 0$, where $W_{AE} > 0$ is the weight of GA. According to the second operation principle, in the meantime there is a generating reaction (GR) applied to E by A, which produces a change rate of $x_E$ as $W_{EA}(x_A - x_E) < 0$, where $W_{EA} > 0$ is the weight of GR. On the other hand, if $x_A > x_E$, the above directions of GA and GR are converse, accordingly.
- Agent A ↔ agent B: Agent A and agent B are also adjacent and generate each other. The GA and GR between A and B yield the change rates $W_{AB}(x_B - x_A)$ and $W_{BA}(x_A - x_B)$ for $x_A$ and $x_B$, respectively.
- Agent A ↔ agent D: Agent A and agent D are spaced apart and thus overcome each other. If $x_D > x_A$, agent D overcomes agent A and causes $x_A$ to decrease. This is the overcoming action (OA) applied to A by D, giving a negative change rate of A as $W_{AD}(x_D - x_A) < 0$, where $W_{AD} < 0$ is the weight of OA. The overcoming reaction (OR) applied to D by A contributes a positive change rate $W_{DA}(x_A - x_D) > 0$ to $x_D$ by noting $W_{DA} < 0$ and $x_D > x_A$. It appears that when agent D overcomes agent A, it increases its own quantity $x_D$ by decreasing the quantity of agent A. On the other hand, if $x_D < x_A$, the above directions of OA and OR are converse, accordingly.
- Agent A ↔ agent C: Agent A and agent C are spaced apart and thus overcome each other. The overcoming action applied to C by A contributes a change rate $W_{CA}(x_A - x_C)$ to $x_C$ with

$W_{CA} < 0$, and the overcoming reaction from C to A contributes a change rate $W_{AC}(x_C - x_A)$ to agent A with $W_{AC} < 0$.

Although the generating (cooperative) and overcoming (antagonistic) interactions between two agents both increase the amount of one agent and decrease the amount of the other agent, the two interactions have opposite effects on the network system. The role of a cooperative interaction causes the agent with larger quantity to decrease its amount, and the agent with smaller quantity to increase its amount so that the quantity gap between the two agents can be reduced. Conversely, the role of an antagonistic interaction causes the agent with larger quantity to increase its amount, and the agent with smaller quantity to decrease its amount so that the quantity gap between the two agents is widened. Therefore, the cooperative interaction tends to promote the balance of the network, while the antagonistic interaction tends to destroy the balance of the network. If all the interactions of a network are cooperative, it is balanced automatically. When there are more and more antagonistic interactions within the network, the network will tend to be unbalanced.

Wuxing Network with five elements has a remarkable property that its cooperative and antagonistic interactions are evenly matched in such a way that each agent is subject to two cooperative interactions from the adjacent agents and two antagonistic interactions from the spaced-apart agents. This symmetry in Wuxing network disappears, if we consider a N-element network with $N \neq 5$.

**Supplementary Table S1** The generating (cooperative) actions and overcoming (antagonistic) actions and their reactions applied to each agent in Wuxing network.

| Agent | Generating action (GA) | Generating reaction (GR) | Overcoming action (OA) | Overcoming reaction (OR) |
|---|---|---|---|---|
| A | $E \to A$ | $B \to A$ | $D \to A$ | $C \to A$ |
| | $W_{AE}(x_E - x_A)$ | $W_{AB}(x_B - x_A)$ | $W_{AD}(x_D - x_A)$ | $W_{AC}(x_C - x_A)$ |
| B | $A \to B$ | $C \to B$ | $E \to B$ | $D \to B$ |
| | $W_{BA}(x_A - x_B)$ | $W_{BC}(x_C - x_B)$ | $W_{BE}(x_E - x_B)$ | $W_{BD}(x_D - x_B)$ |
| C | $B \to C$ | $D \to C$ | $A \to C$ | $E \to C$ |
| | $W_{CB}(x_B - x_C)$ | $W_{CD}(x_D - x_C)$ | $W_{CA}(x_A - x_C)$ | $W_{CE}(x_E - x_C)$ |
| D | $C \to D$ | $E \to D$ | $B \to D$ | $A \to D$ |
| | $W_{DC}(x_C - x_D)$ | $W_{DE}(x_E - x_D)$ | $W_{DB}(x_B - x_D)$ | $W_{DA}(x_A - x_D)$ |
| E | $D \to E$ | $A \to E$ | $C \to E$ | $B \to E$ |
| | $W_{ED}(x_D - x_E)$ | $W_{EA}(x_A - x_E)$ | $W_{EC}(x_C - x_E)$ | $W_{EB}(x_B - x_E)$ |

Like agent A in Fig. 6b, each agent of Wuxing network is subject to four effects: GA, GR, OA, and OR, coming from the other four agents, respectively; meanwhile, each agent in turn applies actions or reactions to the other four agents, as summarized in Supplementary Table S1. Adding the four effects in each row of the table, we obtain the time change rate of each agent as follows:

$$\dot{x}_A = W_{AE}(x_E - x_A) + W_{AB}(x_B - x_A) + W_{AD}(x_D - x_A) + W_{AC}(x_C - x_A), \quad \text{(S1a)}$$

$$\dot{x}_B = W_{BA}(x_A - x_B) + W_{BC}(x_C - x_B) + W_{BE}(x_E - x_B) + W_{BD}(x_D - x_B), \quad \text{(S1b)}$$
$$\dot{x}_C = W_{CB}(x_B - x_C) + W_{CD}(x_D - x_C) + W_{CA}(x_A - x_C) + W_{CE}(x_E - x_C), \quad \text{(S1c)}$$
$$\dot{x}_D = W_{DC}(x_C - x_D) + W_{DE}(x_E - x_D) + W_{DB}(x_B - x_D) + W_{DA}(x_A - x_D), \quad \text{(S1d)}$$
$$\dot{x}_E = W_{ED}(x_D - x_E) + W_{EA}(x_A - x_E) + W_{EC}(x_C - x_E) + W_{EB}(x_B - x_E). \quad \text{(S1e)}$$

Although Eq. (S1) is derived from the operation protocol of Wuxing network, it is actually a general mathematical model for multi-agent network systems. With different settings of the weights $W_{ij}$, we can get different network structures. (A) If $W_{ij} > 0$ for the sides of the pentagon, and $W_{ij} < 0$ for the diagonals of the pentagon, Eq. (S1) represents the model of Wuxing network considered here. (B) If all $W_{ij}$'s are set to be positive, Eq. (S1) becomes the most discussed cooperative network in the literature. (C) If all $W_{ij}$'s are set to be $\pm 1$, Eq. (S1) represents the signed social network, where the positive sign denotes the connection between friends and the negative sign denotes the connection between enemies. (D) If some $W_{ij}$'s are set to zero, Eq. (S1) serves as a model for non-complete graphs that has no connection from element $j$ to element $i$.

**Conservation and Consensus of Wuxing Network.** Wuxing Network has two inherent characteristics. First, its total quantity (total resources) is conserved under the cooperative and antagonistic interactions, and second, its operation tends to allocate the total resources evenly to each agent, that is, the five agents eventually converge to a state of consensus. In other words, the function of Wuxing network is to strike a balance between cooperation and competition when total resources are fixed. The conservation law is a direct result derived from Eq. (5). By adding the five equations in Eq. (5), and noting that the result of adding all the elements of each column in the system matrix $\mathbb{A}$ is zero, we get the following result

$$\frac{d}{dt}(x_A + x_B + x_C + x_D + x_E) = 0, \quad \text{(S2)}$$

which indicates that the summation $x_A(t) + x_B(t) + x_C(t) + x_D(t) + x_E(t)$ is a constant independent of time.

When the stability condition (10) is satisfied, Wuxing network converges to a steady state $X_s = [C_A, C_B, C_C, C_D, C_E]^T$ as shown in Eq. (13), whose existence is guaranteed by the stationary mode $e^{\lambda_1 t}$ with $\lambda_1 = 0$. The eigenvector $V_1$ corresponding to the eigenvalue $\lambda_1 = 0$ satisfies the relation $\mathbb{A}V_1 = \lambda_1 V_1 = 0$. On the other hand, a steady-state solution $X_s$ to Eq. (5) must satisfy the relation $\dot{X}_s = \mathbb{A}X_s = 0$. The above two relations show that the steady-state solution $X_s$ and the eigenvector $V_1$ satisfy the same equation. Hence, the existence of $X_s$ is guaranteed by the existence of $V_1$. An explicit expansion of $\mathbb{A}X_s = \mathbb{A}V_1 = 0$ with $X_s = [C_A, C_B, C_C, C_D, C_E]^T$ leads to the following form

$$\mathbb{A}X_s = \begin{bmatrix} \sigma & b & -d & -c & a \\ a & \sigma & b & -d & -c \\ -c & a & \sigma & b & -d \\ -d & -c & a & \sigma & b \\ b & -d & -c & a & \sigma \end{bmatrix} \begin{bmatrix} C_A \\ C_B \\ C_C \\ C_D \\ C_E \end{bmatrix} = 0, \quad \text{(S3)}$$

whose solution can be found readily as $X_s = \gamma[1, 1, 1, 1,1]^T$ with $\gamma$ being a constant to be determined. Because all the elements in $X_s$ are equal to $\gamma$, we have

$$X_s = [C_A, C_B, C_C, C_D, C_E]^T = [x_A(\infty), x_B(\infty), x_C(\infty), x_D(\infty), x_E(\infty)]^T = \gamma[1, 1, 1, 1,1]^T, \tag{S4}$$

which indicates that all the agents converge to the state of consensus $\gamma$.

The most noteworthy thing is that the consensus γ achieved by Wuxing network has nothing to do with the four weights, and can be determined in advance without the need to solve the differential equations (5). Applying the conservation law (S2) to the initial and steady-state conditions of the network, we have

$$\begin{aligned} &x_A(\infty) + x_B(\infty) + x_C(\infty) + x_D(\infty) + x_E(\infty) \\ &= x_A(0) + x_B(0) + x_C(0) + x_D(0) + x_E(0). \end{aligned} \tag{S5}$$

The combination of the conservation law (S5) with the consensus condition (S4) gives the consensus of Wuxing network as

$$\gamma = \big(x_A(0) + x_B(0) + x_C(0) + x_D(0) + x_E(0)\big)/5. \tag{S6}$$

This result shows that according to its operation protocol, Wuxing network can drive the five agents to the consensus $\gamma$, which is always equal to the average of their initial values, regardless of the settings of the weights. From the viewpoint of resource allocation, Wuxing network operating in the state of consensus tends to allocate its total resources evenly to the five agents.

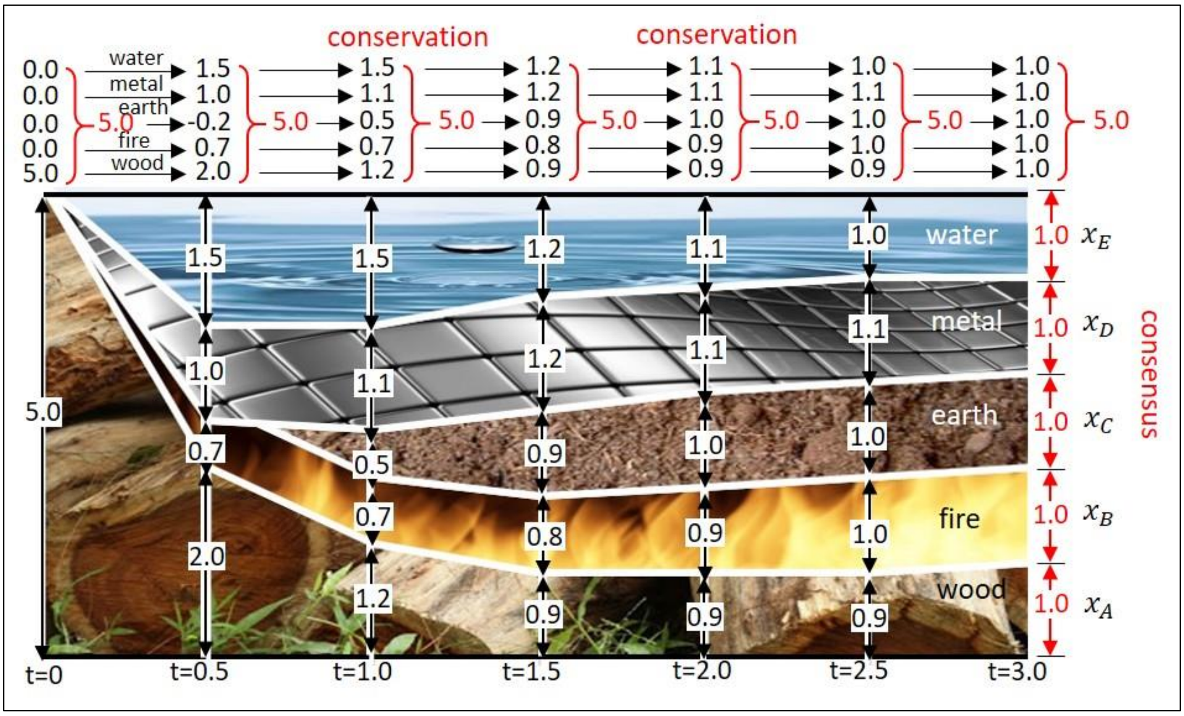


**Supplementary Fig. S2 The stacked area chart showing the conservation (horizontal direction) and consensus (vertical direction) in Wuxing network.** The displayed value of $x_i(t)$ is solved from Eq. (5) with initial conditions $x_A(0) = 5$, $x_B(0) = x_C(0) = x_D(0) = x_E(0) = 0$ and with weights $a + b = 9/2$, $a - b = 1$, $c + d = 1$ and $c - d = -1/2$. The conservation property shows that the total quantity of $x_i(t)$ is equal to 5 at any moment. The consensus property shows that operation of Wuxing network tends to allocate the total quantity evenly to the five agents.

The conservation law of Wuxing network is demonstrated numerically in terms of the stacked area chart as shown in Supplementary Fig. S2, where the initial conditions are set to $x_A(0) = 5$ and $x_B(0) = x_C(0) = x_D(0) = x_E(0) = 0$ with weights $a_+ = 9/2$, $a_- = 1$, $c_+ = -1$ and $c_- = 1/2$. The time evolution of the quantitative indices $x_i(t)$ solved from Eq. (5) shows that at each moment, the sum of $x_i(t)$ is always equal to 5, which is the total quantity evaluated at the initial condition. Also shown in Supplementary Fig. S2 is the state of consensus achieved by Wuxing network. It can be seen that all the agents eventually converge to the same value $x_i = 1$, which is exactly the result of distributing the total quantity evenly to each agent.

From the viewpoint of resource allocation, Wuxing network operating in the state of consensus tends to allocate its total resources evenly to the five agents. Supplementary Fig. S3 illustrates the allocation process of Wuxing network starting from the initial condition $x_A(0) = 5$, $x_B(0) = x_C(0) = x_D(0) = x_E(0) = 0$, and eventually approaching the state of consensus $x_A(\infty) = x_B(\infty) = x_C(\infty) = x_D(\infty) = x_E(\infty) = 1$.

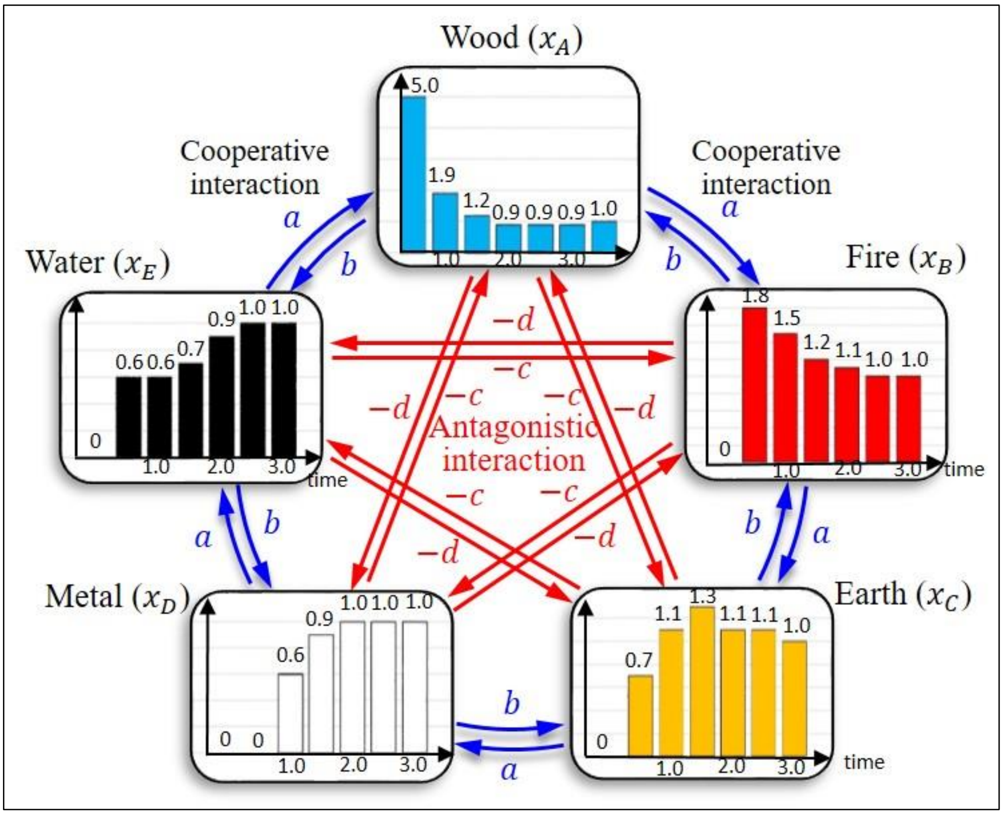


**Supplementary Fig. S3 Wuxing network operating in the state of consensus.** The bar graphs is obtained by solving Eq. (5) with the same conditions used in Supplementary Fig. S2 to demonstrate the time evolution of Wuxing network, which starts from the initial condition $x_A(0) = 5$, $x_B(0) = x_C(0) = x_D(0) = x_E(0) = 0$, and eventually arrives at the state of consensus $x_A(\infty) = x_B(\infty) = x_C(\infty) = x_D(\infty) = x_E(\infty) = 1$. The results indicates that Wuxing network operating in the state of consensus has an internal mechanism of allocating the total resources evenly to each agent.

**The Derivation of General Golden Ratio.** The role of the general golden ratio $\varphi_N$ in the general Wuxing network is just the role of $\varphi$ in Wuxing network. If we replace $\varphi$ by $\varphi_N$, all the previous

results derived for Wuxing network can be extended directly to the general Wuxing network. For a four-element network (see Fig. 5), there is only one diagonal connecting to the vertex $P_1$, and its length is $l_1 = \sqrt{2}s$. Substituting $N = 4$ and $l_1 = \sqrt{2}s$ into Eq. (24), we obtain $a/c > 1$, which is the stability condition for the four-element network. This condition can be confirmed by the eigenvalues of the system matrix $\mathbb{A}_4$ in Eq. (22) with $N = 4$:

$$\lambda_1 = 0, \qquad \lambda_2 = -4a, \qquad \lambda_{3,4} = -2a + 2c. \tag{S7}$$

Network's stability requires that the largest non-zero eigenvalue must be negative, i.e., $\lambda_{3,4} = -2a + 2c < 0$, which gives the same result $a/c > 1$ as derived from Eq. (24).

For a six-element network, there are three diagonals connecting to the vertex $P_1$, the lengths of which are $l_2 = 2s$, $l_1 = l_3 = \sqrt{3}s$. Using these data in Eq. (24) yields

$$\frac{a}{c} > \frac{1}{2}\sum_{k=1}^{3}\left(\frac{l_k}{s}\right)^2 = \frac{1}{2}\left(\left(\frac{2s}{s}\right)^2 + \left(\frac{\sqrt{3}s}{s}\right)^2 + \left(\frac{\sqrt{3}s}{s}\right)^2\right) = 5. \tag{S8}$$

This is the stability condition for the six-element network. This condition can be confirmed by the eigenvalues of the system matrix $\mathbb{A}_6$ in Eq. (22) with $N = 6$:

$$\lambda_1 = 0,\ \lambda_2 = -2(2a - c),\ \lambda_{3,4} = -3(a - c),\ \lambda_{5,6} = -(a - 5c). \tag{S9}$$

The largest non-zero eigenvalue is $\lambda_{5,6}$ and the stability condition $\lambda_{5,6} < 0$ gives $a > 5c$, the same as Eq. (S8).

For an eight-element network, there are five diagonals connecting to the vertex $P_1$, the lengths of which are

$$l_3 = s\sqrt{4 + 2\sqrt{2}}\ ,\ \ l_2 = l_4 = s\left(1 + \sqrt{2}\right),\ \ l_1 = l_5 = s\sqrt{2 + \sqrt{2}}. \tag{S10}$$

Using these data in Eq. (24) yields

$$\frac{a}{c} > \frac{1}{2}\sum_{k=1}^{5}\left(\frac{l_k}{s_1}\right)^2 = \frac{1}{2}\left[4 + 2\sqrt{2} + 2\left(1 + \sqrt{2}\right)^2 + 2(2 + \sqrt{2})\right] = 7 + 4\sqrt{2}. \tag{S11}$$

This is the stability condition for the eight-element network. This condition again can be confirmed by the eigenvalues of the system matrix $\mathbb{A}_8$ in Eq. (22):

$$\lambda_1 = 0,\ \lambda_2 = -4(a - c), \qquad \lambda_{3,4} = -2(a - 3c),$$
$$\lambda_{5,6} = -\left(2 - \sqrt{2}\right)a + \left(6 + \sqrt{2}\right)c, \qquad \lambda_{7,8} = -\left(2 + \sqrt{2}\right)a + \left(6 - \sqrt{2}\right)c. \tag{S12}$$

The largest non-zero eigenvalue is $\lambda_{7,8}$ and the stability condition $\lambda_{7,8} < 0$ gives

$$\lambda_{7,8} = -\left(2 - \sqrt{2}\right)a + \left(6 + \sqrt{2}\right)c < 0 \ \ \Rightarrow \ \ \frac{a}{c} > \frac{6 + \sqrt{2}}{2 - \sqrt{2}} = 7 + 4\sqrt{2}, \tag{S13}$$

which is identical to Eq. (S11) obtained from the geometrical condition (24).

The geometric inequality provided by Eq. (24) allows us to quickly judge the stability of the N-element network without computing the eigenvalues of the system matrix $\mathbb{A}_N$. But when the value of N is large, calculating the sum of squares of all diagonals is still a troublesome task. There is a very simple formula for computing $\varphi_N^2$, in which the stability condition of the N-element network

can be judged directly by the value of N, and there is no need to calculate the diagonals of the N-sided polygon.

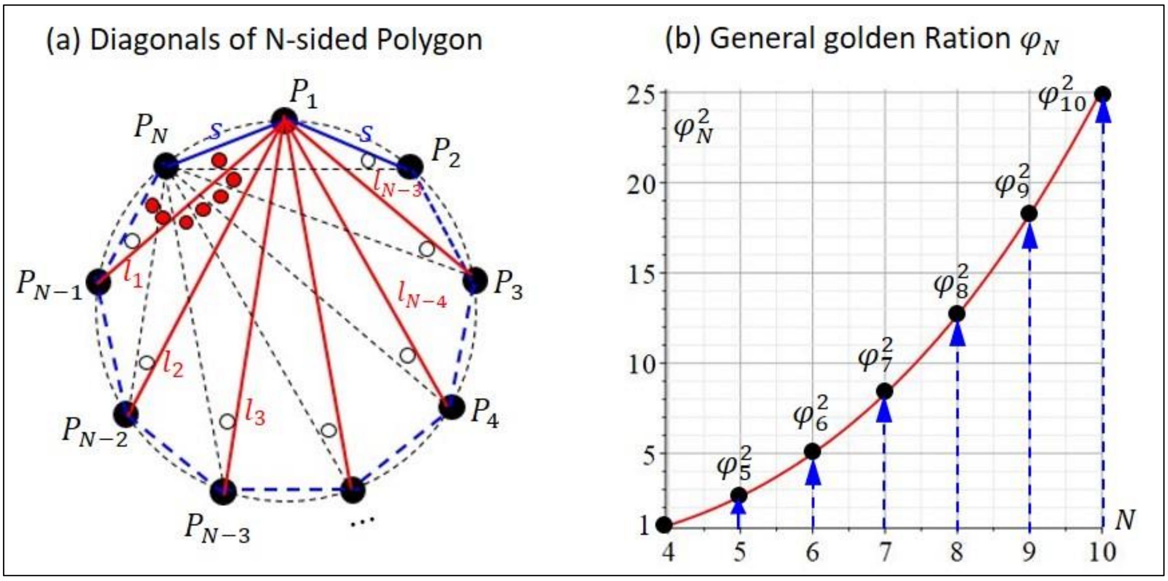


**Supplementary Fig. S4 The N-sided polygon and the related general golden ratio $\varphi_N$.** (a) There are $N-3$ diagonals, $l_1$, $l_2$, $\cdots$, $l_{N-3}$, with antagonistic connecting to vertex $P_1$ of the N-sided polygon, but only two sides with cooperative connecting to $P_1$. As N increases, the number of antagonistic connections increases, but the number of cooperative connections remains at two. (b) The general golden ratio defined as $\varphi_N^2 = (l_1^2 + l_2^2 + \cdots + l_{N-3}^2)/(2s^2)$ is plotted as a function of N, where $\varphi_5$ is the conventional golden ratio. The balance condition for the N-element Wuxing network is given by $a/c = \varphi_N^2$, showing that the ratio of the cooperative weight $a$ to the antagonistic weight $c$ must increase with N to balance the increasing antagonistic interaction contributed by the increasing number of diagonals connecting to $P_1$.

Referring to Supplementary Fig. S4a, $P_1, P_2, \cdots, P_N$, are the N vertices of a regular N polygon, and the internal angle of each vertex is $(N-2)\pi/N$. The angles indicated by the small red and white circles in Fig. S4a are all equal to $\pi/N$, because they are the circumference angles corresponding to arcs of the same length. Now we take $P_1$ as the starting point and connect it to other vertices to form a total of $N-3$ diagonals, $l_1, l_2, \cdots, l_{N-3}$. Applying the Law of Sines sequentially to the triangles containing the side of $P_1P_N$ and the diagonal $l_i$, we get the following ratios

$$\frac{l_i}{s} = \frac{\sin\big((N-1-i)\pi/N\big)}{\sin(\pi/N)}, \qquad i = 1, 2, \cdots, N-3. \tag{S14}$$

Substituting the above $N-3$ diagonals $l_i$ into Eq. (24), the condition for the stability of the N-element network becomes

$$\frac{a}{c} > \varphi_N^2 = \frac{1}{2}\sum_{k=2}^{N-2} \frac{\sin^2(k\pi/N)}{\sin^2(\pi/N)} = \frac{1/4}{\sin^2(\pi/N)} \sum_{k=2}^{N-2} \left(1 - \cos\left(\frac{2k\pi}{N}\right)\right). \tag{S15}$$

To sum the above cosine functions, we note the following identity

$$\sum_{k=1}^{N} \cos\left(k \cdot \frac{2\pi}{N}\right) = 0. \tag{S16}$$

Using this identity in Eq. (S15), we obtain the main result

$$\frac{a}{c} > \varphi_N^2 = \frac{1/4}{\sin^2(\pi/N)}\left[N - 2 + 2\cos\left(\frac{2\pi}{N}\right)\right] = \frac{N}{4}\csc^2\left(\frac{\pi}{N}\right) - 1. \tag{S17}$$

Similar to the five-element Wuxing network, we can show that if $a/c > \varphi_N^2$, the N-element network is stable and reaches the state of consensus as $t \to \infty$; if $a/c = \varphi_N^2$, the N-element network is balanced and exhibits harmonic oscillation, and if $a/c < \varphi_N^2$, the N-element network is unstable. For the case of $N = 5$ corresponding to Wuxing network, we have $\varphi_N^2 = \varphi^2$, and Eq. (S17) recovers Eq. (10) with $a = b$ and $c = d$. All the other special cases of $N$ considered above can be reconfirmed by the simple rule given by Eq. (S17). With increasing $N$, the number of agents connecting diagonally to $P_1$ increases, but there are always only two agents, i.e., $P_2$ and $P_N$, that are adjacent to $P_1$. Therefore, in order to balance the increasing antagonistic interaction, the ratio of the cooperative weight $a$ to the antagonistic weight $c$ must increase with increasing $N$, as required by Eq. (S17).

In Supplementary Fig. S4b, the increasing trend of $\varphi_N^2$ with $N$ is similar to the behavior of a polynomial function. This is because when $N$ is large, $\varphi_N^2$ can be well approximated by a cubic polynomial function of $N$ as:

$$\varphi_N^2 = \frac{N}{4}\csc^2\left(\frac{\pi}{N}\right) - 1 \approx \frac{N^3}{4\pi^2} + \frac{N}{12} - 1, \quad N \geq 4. \tag{S18}$$

Even for the minimum value of $N$, i.e., $N = 4$, the largest error between the two expressions is only 4.55%. Eq. (S18) provides a simple polynomial formula to determine the ratio $a/c$ required to ensure the stability for any N-element Wuxing network.
